# Inhibition of activin A receptor signalling attenuates age-related pathological cardiac remodelling

**DOI:** 10.1242/dmm.049424

**Published:** 2022-05-09

**Authors:** Nicolas G. Clavere, Ali Alqallaf, Kerry A. Rostron, Andrew Parnell, Robert Mitchell, Ketan Patel, Samuel Y. Boateng

**Affiliations:** 1 Institute of Cardiovascular and Metabolic Research, School of Biological Sciences, Health and Life Sciences Building, University of Reading, Whiteknights, Reading RG6 6UB, UK; 2 Centre for Inflammatory Disease, Department of Immunology and Inflammation, Imperial College London, Commonwealth Building, Hammersmith Hospital, Du Cane Road, London W12 0NN, UK

**Keywords:** Activin signalling, Ageing, DNA repair, Oxidative stress, Heart

## Abstract

In the heart, ageing is associated with DNA damage, oxidative stress, fibrosis and activation of the activin signalling pathway, leading to cardiac dysfunction. The cardiac effects of activin signalling blockade in progeria are unknown. This study investigated the cardiac effects of progeria induced by attenuated levels of *Ercc1*, which is required for DNA excision and repair, and the impact of activin signalling blockade using a soluble activin receptor type IIB (sActRIIB). DNA damage and oxidative stress were significantly increased in *Ercc1^Δ^*^/−^ hearts, but were reduced by sActRIIB treatment. sActRIIB treatment improved cardiac systolic function and induced cardiomyocyte hypertrophy in *Ercc1^Δ^*^/−^ hearts. RNA-sequencing analysis showed that in *Ercc1^Δ/−^* hearts, there was an increase in pro-oxidant and a decrease in antioxidant gene expression, whereas sActRIIB treatment reversed this effect. *Ercc1^Δ/−^* hearts also expressed higher levels of anti-hypertrophic genes and decreased levels of pro-hypertrophic ones, which were also reversed by sActRIIB treatment. These results show for the first time that inhibition of activin A receptor signalling attenuates cardiac dysfunction, pathological tissue remodelling and gene expression in *Ercc1*-deficient mice and presents a potentially novel therapeutic target for heart diseases.

## Abstract (precis)

**Summary:** Attenuated DNA repair is associated with pathological cardiac remodelling and gene expression. Much of this phenotype is attenuated by inhibition of the activin signalling pathway using soluble activin receptor treatment.

## INTRODUCTION

Ageing is characterised by a progressive loss of physiological integrity leading to impaired organ function and increased risk of death. Disruption to tissue homeostasis is a major cause of diseases such as cardiovascular diseases (North and Sinclair, 2012), cancer (Niccoli and Partridge, 2012) and neurological disorders (Licher et al., 2019). Ageing is the consequence of the impairment of multiple interconnected pathways such as protein turnover, disregulation of signalling pathways, DNA repair and telomere attrition. These failing mechanisms lead to an accumulation of damage at the cellular level with an associated increase in oxidative stress and inflammation (López-Otín et al., 2013).

Cardiomyopathies such as heart failure are a major public health issue, with a worldwide prevalence of over 23 million (Ambrosy et al., 2014). Ageing significantly increases risk factors such as coronary artery disease (Jenča et al., 2021), hypertension (Hansson, 1998) and metabolic disorders such as obesity (Kenchaiah et al., 2002; Tune et al., 2017). These disorders are characterised by pathological remodelling leading to cardiac dysfunction, hypertrophy (Lazzeroni et al., 2016), inflammation (Murphy et al., 2020), fibrosis (Querejeta et al., 2004) and gene expression.

Although normal ageing is slow and progressive, there are a number of genetic mutations that significantly increase the rate of ageing, in a condition called progeria. The progeroid syndrome can be induced by mutations in the nuclear structural protein lamin A leading to Hutchinson–Gilford progeria syndrome (HGPS) (Ahmed et al., 2018). Defects in DNA repair and replication systems also result in progeria leading to Werner syndrome (WS) (Oshima et al., 1993) and Bloom syndrome (Arora et al., 2014). A defect in the nucleotide excision repair system due to a mutation in the *Ercc1-Xpf* endonuclease system leads to several human disorders such as Cockayne syndrome and Xeroderma pigmentosum (Karikkineth et al., 2017), Fanconi anaemia (Kashiyama et al., 2013) and cerebro-oculo-facio-skeletal syndrome (Jaspers et al., 2007). Cardiovascular diseases are the main cause of death in progeroid syndromes (Ahmed et al., 2018; Oshima et al., 1993; Crome and Kanjilal, 1971; Yuen et al., 2001). In HGPS, children suffer from severe atherosclerosis with a 50% loss of vascular smooth muscle cells in the media of the aorta and an increase of the adventitia thickness due to increased fibrosis (Olive et al., 2010). Mice with WS display increased plasma cholesterol, triglycerides, insulin blood levels and interstitial cardiac fibrosis due to elevated levels of oxidative stress (Massip et al., 2006). In *Ercc1^Δ/−^* progeroid mice, the enzymatic activity of the endonuclease is reduced to 5% (Gurkar and Niedernhofer, 2015). An impaired DNA repair system as a result of *Ercc1* deficiency was found to lead to a systemic ageing process due to the accumulation of DNA damage, leading to a maximum lifespan of 24 weeks (Niedernhofer et al., 2006; Dollé et al., 2011). *Ercc1* deficiency also leads to sarcopenia (Alyodawi et al., 2019) and neurological disorders (Sepe et al., 2016; Sharma et al., 2018). Importantly, potential effects of *Ercc1* deficiency on the heart have not been extensively determined. A previous study has shown that the *Ercc1^Δ/−^* progeroid mouse hearts are smaller than controls from week 1 of age. The *Ercc1^Δ/−^* hearts decreased in weight from week 10, whereas control hearts continued to increase (Dolle et al., 2011). This suggests that the onset of progeria is associated with significant cardiac changes that have yet to be fully characterised.

It has been shown that circulating levels of activin A were increased in patients with heart failure (Yndestad et al., 2004). Circulating levels of activin A and myostatin were shown to have negative impacts on cardiac tissue physiology through binding to activin receptor type IIB (encoded by *ACVR2B*) (Loumaye et al., 2015). Soluble activin receptor type IIB (sActRIIB) has been used experimentally as a ligand trap to block receptor signalling with beneficial effects on cardiac muscle in heart failure (Lee et al., 2005). The sActRIIB treatment also showed beneficial effects during normal cardiac ageing by preserving cardiac function (Roh et al., 2019). We have previously shown that sActRIIB treatment of *Ercc1^Δ/−^* mice significantly improves muscle mass, tone and function (Alyodawi et al., 2019). In this study, we investigated whether sActRIIB treatment of *Ercc1^Δ/−^* mice could improve pathological cardiac remodelling and contractile function associated with progeria.

## RESULTS

### The *Ercc1^Δ/−^* progeroid mouse phenotype

**Fig. 1.**
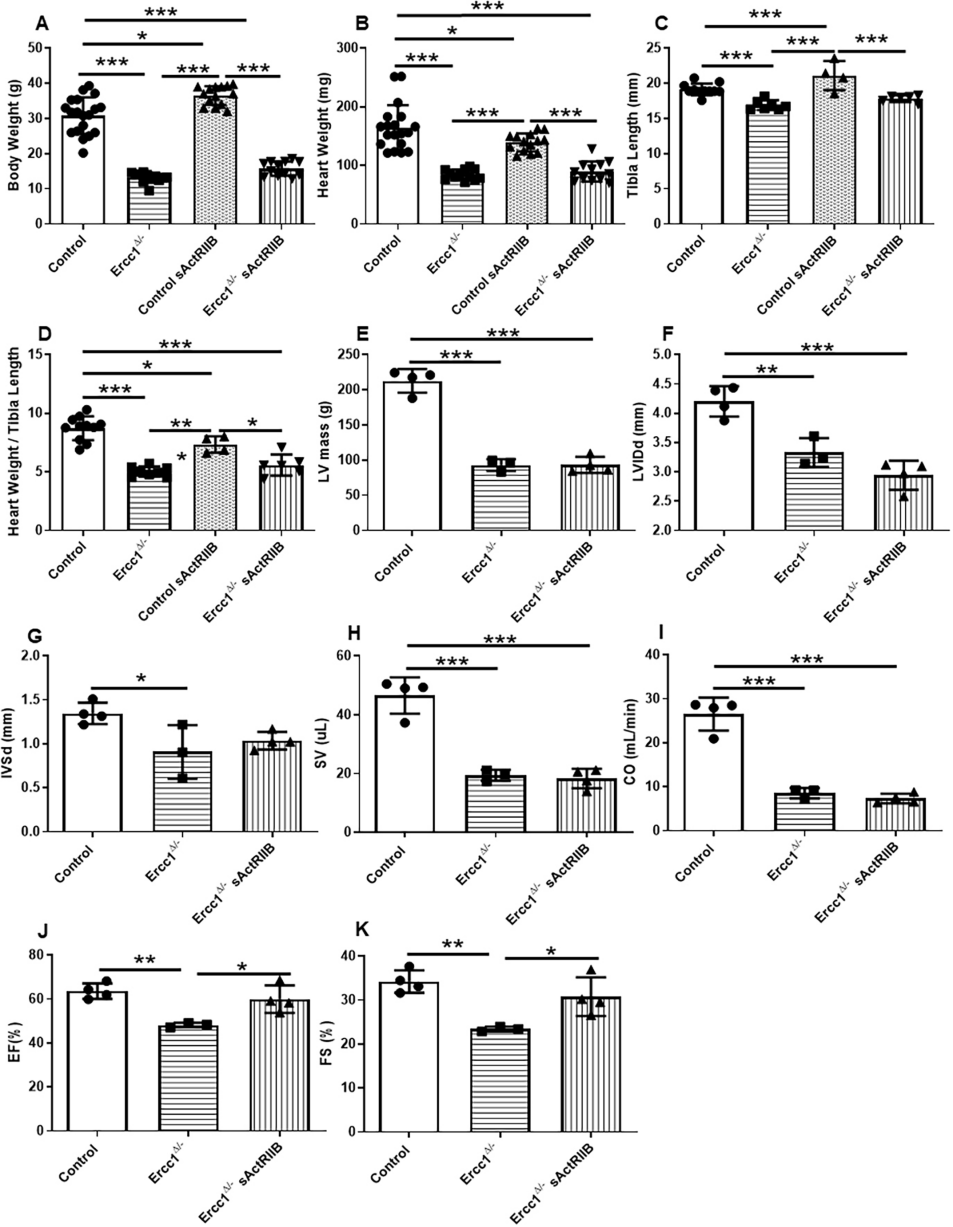
**Phenotypic features of control and *Ercc1^Δ/−^* mice.** (A) Body weight was measured for control mice (*n*=19), *Ercc1^Δ/−^* sActRIIB mice (*n*=12), *Ercc1^Δ/−^* mice (*n*=12) and control sActRIIB mice (*n*=14). Control versus *Ercc1^Δ/−^*, *P*<0.000001; control versus control sActRIIB, *P*=0.000123; control versus *Ercc1^Δ/−^* sActRIIB, *P*<0.000001; *Ercc1^Δ/−^* versus control sActRIIB, *P*<0.000001; control sActRIIB versus *Ercc1^Δ/−^* sActRIIB, *P*<0.000001. (B) Heart weight was measured from control mice (*n*=19), *Ercc1^Δ/−^* sActRIIB mice (*n*=12), *Ercc1^Δ/−^* mice (*n*=12) and control sActRIIB mice (*n*=14). Control versus *Ercc1^Δ/−^*, *P*<0.000001; control versus control sActRIIB, *P*=0.035282; control versus *Ercc1^Δ/−^* sActRIIB, *P*<0.000001; *Ercc1^Δ/−^* versus control sActRIIB, *P*=0.000006; control sActRIIB versus *Ercc1^Δ/−^* sActRIIB, *P*=0.000036. (C) Tibia length was measured from control mice (*n*=11), *Ercc1^Δ/−^* sActRIIB mice (*n*=6), *Ercc1^Δ/−^* mice (*n*=7) and control sActRIIB mice (*n*=4). Control versus *Ercc1^Δ/−^*, *P*=0.000507; control versus control sActRIIB, *P*=0.012632; *Ercc1^Δ/−^* versus control sActRIIB, *P*=0.000003; control sActRIIB versus *Ercc1^Δ/−^* sActRIIB, *P*<0.000181. (D) Heart weight to tibia length ratio was measured from heart weight and tibia length measurements from control mice (*n*=11), *Ercc1^Δ/−^* sActRIIB mice (*n*=6), *Ercc1^Δ/−^* mice (*n*=7) and control sActRIIB mice (*n*=4). Control versus *Ercc1^Δ/−^*, *P*<0.000001; control versus control sActRIIB, *P*=0.038378; control versus *Ercc1^Δ/−^* sActRIIB, *P*<0.000001; *Ercc1^Δ/−^* versus control sActRIIB, *P*=0.000687; control sActRIIB versus *Ercc1^Δ/−^* sActRIIB, *P*=0.015465. (E-J) Cardiac function of the control and *Ercc1^Δ/−^* mice. (E) Left ventricle (LV) mass was measured from control mice (*n*=4), *Ercc1^Δ/−^* sActRIIB mice (*n*=4), *Ercc1^Δ/−^* mice (*n*=3). Control versus *Ercc1^Δ/−^*, *P*<0.0001; control versus *Ercc1^Δ/−^* sActRIIB, *P*<0.0001. (F) Left ventricle internal diameter end of diastole (LVIDd) was measured from control mice (*n*=4), *Ercc1^Δ/−^* sActRIIB mice (*n*=4), *Ercc1^Δ/−^* mice (*n*=3). Control versus *Ercc1^Δ/−^*, *P*=0.0047; control versus *Ercc1^Δ/−^* sActRIIB, *P*=0.0003. (G) Interventricular septum thickness in diastole (IVsd) was measured from control mice (*n*=4), *Ercc1^Δ/−^* sActRIIB mice (*n*=4), *Ercc1^Δ/−^* mice (*n*=3). Control versus *Ercc1^Δ/−^*, *P*=0.0317. One-way ANOVA parametric test was realised followed by a Tukey post-hoc test to compare the mean for different groups. (H) Stroke volume (SV) was measured from control mice (*n*=4), *Ercc1^Δ/−^* sActRIIB mice (*n*=4), *Ercc1^Δ/−^* mice (*n*=3). Control versus *Ercc1^Δ/−^*, *P*=0.0001; control versus *Ercc1^Δ/−^* sActRIIB, *P*<0.0001. (I) Cardiac output (CO) was measured from control mice (*n*=4), *Ercc1^Δ/−^* sActRIIB mice (*n*=4), *Ercc1^Δ/−^* mice (*n*=3). Control versus *Ercc1^Δ/−^*, *P*<0.0001; control versus *Ercc1^Δ/−^* sActRIIB, *P*<0.0001. (J) Ejection fraction (EF) was measured from control mice (*n*=4), *Ercc1^Δ/−^* sActRIIB mice (*n*=4), *Ercc1^Δ/−^* mice (*n*=3). Control versus *Ercc1^Δ/−^*, *P*=0.0045; *Ercc1^Δ/−^* sActRIIB versus *Ercc1^Δ/−^* sActRIIB, *P*=0.0197. (K) Fractional shortening (FS) was measured from control mice (*n*=4), *Ercc1^Δ/−^* sActRIIB mice (*n*=4), *Ercc1^Δ/−^* mice (*n*=3). Control versus *Ercc1^Δ/−^*, *P*=0.0049; *Ercc1^Δ/−^* sActRIIB versus *Ercc1^Δ/−^* sActRIIB, *P*=0.0357. All results are expressed as mean±s.d. *P≤0.05, **P<0.01, ***P<0.001. A one-way ANOVA parametric test was followed by a Tukey post-hoc test to compare the mean from different groups.

To determine the phenotype of the *Ercc1^Δ/−^* progeroid mice with and without sActRIIB treatment, body weight, heart weight and tibia length were measured in the four experimental groups. The body weight of *Ercc1^Δ/−^* mice was decreased by 57% in comparison to control mice (*P*<0.000001) (Fig. 1A). sActRIIB had no impact on the body weight of *Ercc1^Δ/−^* mice. The body weight of control mice increased by 16% following sActRIIB treatment (*P*<0.000123).

The heart weight of *Ercc1^Δ/−^* mice was reduced by 49% in comparison to control mice at 16 weeks (*P*<0.000001). sActRIIB treatment had no impact on *Ercc1^Δ/−^* hearts. However, sActRIIB treatment decreased the heart weight in control mice (*P*<0.035282) (Fig. 1B).

The tibia length of *Ercc1^Δ/−^* mice was reduced by 12% in comparison to control mice at 16 weeks (*P*<0.000507). sActRIIB had no impact on the tibia length of *Ercc1^Δ/−^* mice. However, sActRIIB-treated control mice showed a 9% increase in tibia length (*P*<0.012632) (Fig. 1C).

The heart weight to tibia length ratio gives an indication of cardiac size relative to growth (Yin et al., 1982). The heart weight to tibia length ratio was reduced by 42% in *Ercc1^Δ/−^* mice compared with control mice (*P*<0.000001) (Fig. 1D). A similar result was observed for treatment with the soluble activin receptor. sActRIIB treatment of *Ercc1^Δ/−^* mice had no significant impact on the heart weight to tibia length ratio. However, sActRIIB treatment of control mice showed a 16% decrease of heart weight to tibia length ratio (*P*<0.038378). These data were also expressed as a ratio of heart weight to body weight (Fig. S1).

### Cardiac contractile function

To determine cardiac function in *Ercc1^Δ/−^* progeroid mice following sActRIIB treatment, mice were assessed at 12 and 16 weeks and the results were averaged (for control, *Ercc1^Δ/−^* and *Ercc1^Δ/−^* sActRIIB cohorts). The left ventricle mass was assessed by ultrasound for *Ercc1^Δ/−^* and control mice and was decreased by 56% in comparison to the control hearts (*P*<0.0001) (Fig. 1E). sActRIIB had no impact on left ventricular mass.

The left ventricle internal diameter at the end of diastole for *Ercc1^Δ/−^* mice was reduced by 20% in comparison to the control mice (*P*=0.0047) (Fig. 1F). The left ventricle internal diameter at the end of diastole for *Ercc1^Δ/−^* sActRIIB mice was reduced by 30% in comparison to the control mice (*P*=0.0003). The interventricular septum thickness in diastole of *Ercc1^Δ/−^* mice was reduced by 32% in comparison to the control mice (*P*=0.0317) (Fig. 1G), but was not altered by sActRIIB treatment.

The stroke volume for *Ercc1^Δ/−^* mice was reduced by 58% in comparison to the control (*P*=0.0001) (Fig. 1H), a feature not changed by sActRIIB treatment. Cardiac output was reduced by 68% *Ercc1^Δ/−^* mice, but was not altered by sActRIIB treatment (Fig. 1I).

Next, we examined systolic function. The ejection fraction of *Ercc1^Δ/−^* mouse hearts was reduced by 24% in comparison to control, whereas sActRIIB treatment significantly increased it (Fig. 1J). There was no significant difference in the ejection fraction between control and *Ercc1^Δ/−^* sActRIIB hearts. The fractional shortening of *Ercc1^Δ/−^* mice was reduced by 31% in comparison to the control mice, and sActRIIB treatment significantly increased it (Fig. 1K). There was no significant difference in the fractional shortening between control and *Ercc1^Δ/−^* sActRIIB hearts.

### Activin blockade decreases cardiac fibrosis

**Fig. 2.**
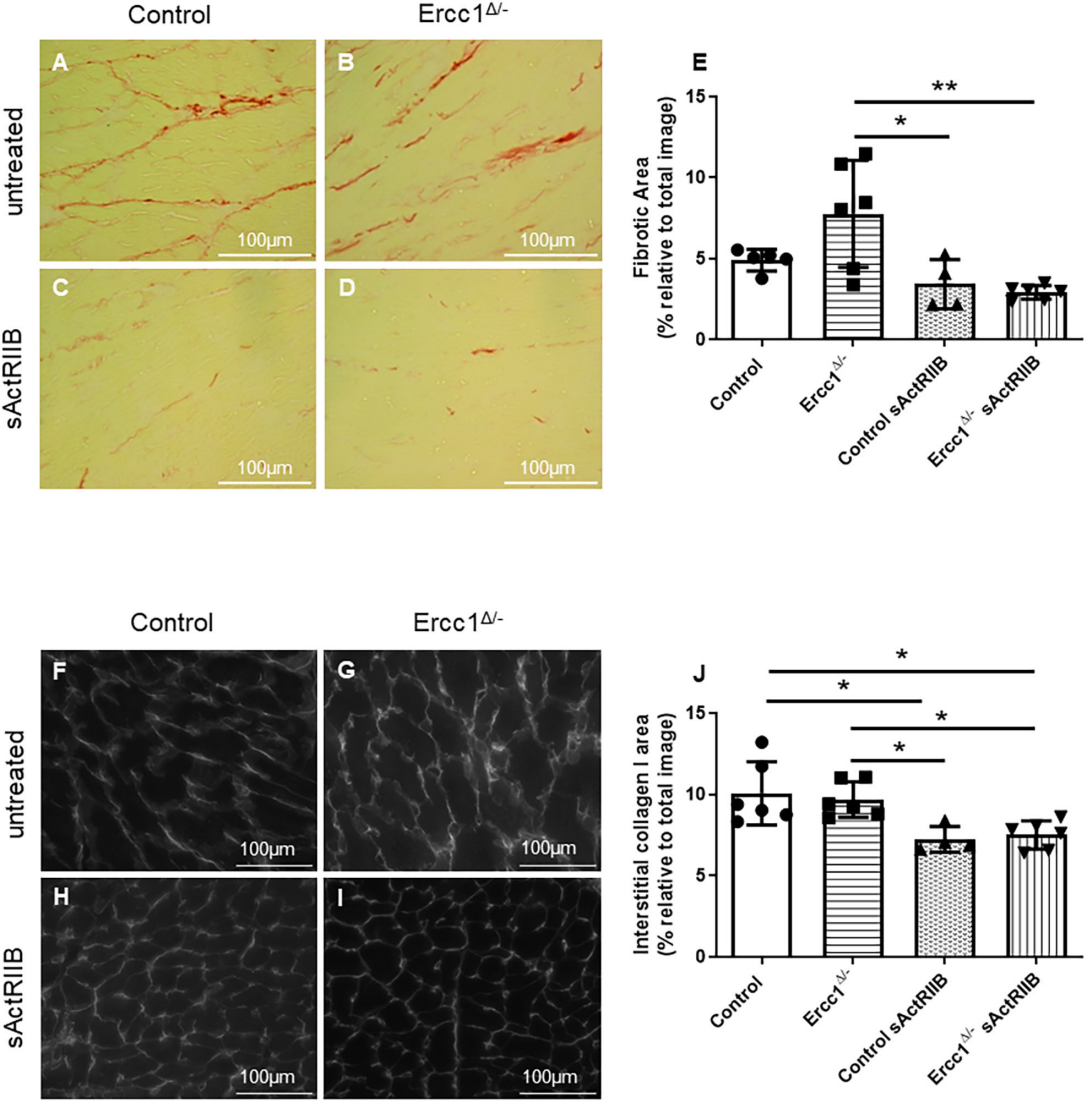
**sActRIIB treatment decreases cardiac interstitial collagen in control and *Ercc1^Δ/−^* treated mice.** (A-D) Picrosirius Red staining highlights the cardiac fibrosis from control and *Ercc1^Δ/−^* mice. Cardiac frozen sections from untreated control mice (*n*=5) (A), untreated *Ercc1^Δ/−^* mice (*n*=6) (B), sActRIIB-treated control mice (*n*=4) (C) and sActRIIB-treated *Ercc1^Δ/−^* mice (*n*=6) (D) aged 16 weeks. (E) Fibrotic area was quantified with ImageJ software, expressed as a percentage relative to the total picture and then averaged and pooled for each animal. Control versus *Ercc1^Δ/−^*, *P*=0.109335; control versus control sActRIIB, *P*=0.669098; *Ercc1^Δ/−^* versus control sActRIIB, *P*=0.014217; *Ercc1^Δ/−^* versus *Ercc1^Δ/−^* sActRIIB, *P*=0.002349. (F-I) Collagen I immunostaining highlights the cardiac interstitial collagen I deposit around the left ventricle from control and *Ercc1^Δ/−^* mice. Cardiac frozen sections from untreated control mice (*n*=6) (F), untreated *Ercc1^Δ/−^* mice (*n*=6) (G), sActRIIB-treated control mice (*n*=4) (H) and sActRIIB-treated *Ercc1^Δ/−^* mice (*n*=6) (I) aged 16 weeks. (J) Collagen area was quantified with ImageJ software then averaged and pooled for each animal. Control versus *Ercc1^Δ/−^*, *P*=0.955188; control versus control sActRIIB, *P*=0.016173; control versus *Ercc1^Δ/−^* sActRIIB, *P*=0.015384; *Ercc1^Δ/−^* versus control sActRIIB, *P*=0.041631; *Ercc1^Δ/−^* versus *Ercc1^Δ/−^* sActRIIB, *P*=0.044248. One-way ANOVA parametric test was followed by a Tukey post-hoc test to compare the mean from different groups. Results are expressed as mean±s.d. *P≤0.05, **P<0.01. Scale bars: 100 μm.

Picrosirius Red staining was performed to visualise the cardiac fibrosis. The results showed that there was no change in *Ercc1^Δ/−^* hearts relative to control hearts. However, *Ercc1^Δ/−^* mice treated with sActRIIB showed decreased fibrosis (*P*<0.002349) (Fig. 2A-E). Collagen I staining was also performed by immunostaining since it makes up 80% of the cardiac extracellular matrix (ECM). As with Picrosirius Red staining, there was no change in collagen I in *Ercc1^Δ/−^* mice compared with control mice. However, sActRIIB treatment resulted in a significant decrease in both control and *Ercc1^Δ/−^* mouse hearts, by 28% (*P*<0.016173) and 22% (*P*<0.044248), respectively (Fig. 2F-J). These changes were associated with a significant increase in tissue interstitial space in *Ercc1^Δ/−^* mice compared with control mice (Fig. S2).

### Activin blockade decreases DNA damage and oxidative stress in *Ercc1^Δ/−^* mice

**Fig. 3.**
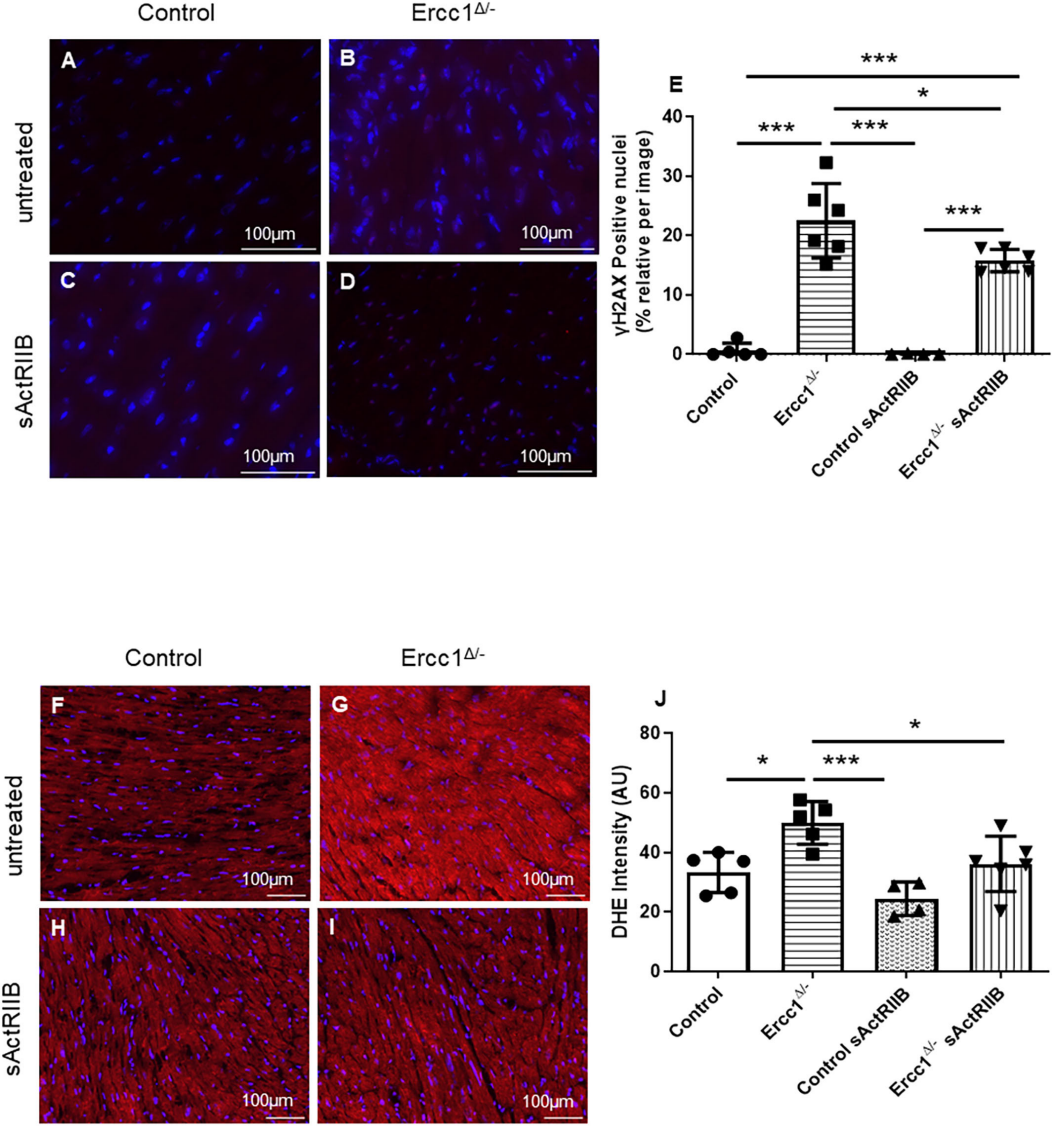
**DNA damage is increased in *Ercc1^Δ/−^* progeroid mouse hearts but reduced by sActRIIB treatment.** (A-D) γH2AX staining highlights the DNA damage in the left ventricle from control and *Ercc1^Δ/−^* mice. Cardiac frozen sections from untreated control mice (*n*=5) (A), untreated *Ercc1^Δ/−^* mice (*n*=6) (B), sActRIIB-treated control mice (*n*=4) (C) and sActRIIB-treated *Ercc1^Δ/−^* mice (*n*=6) (D) aged 16 weeks. (E) γH2AX-positive nuclei were counted using ImageJ software, and then each count was normalized to the count of the total nuclei for each picture. One-way ANOVA parametric test was performed followed by a Tukey post-hoc test to compare the mean from different groups. Results are expressed as mean of positive nuclei±s.d. Control versus *Ercc1^Δ/−^*, *P*<0.000001; control versus control sActRIIB, *P*=0.994267; control versus *Ercc1^Δ/−^* sActRIIB, *P*=0.000013; *Ercc1^Δ/−^* versus control sActRIIB, *P*<0.000001; *Ercc1^Δ/−^* versus *Ercc1^Δ/−^* sActRIIB, *P*=0.023025; control sActRIIB versus *Ercc1^Δ/−^* sActRIIB, *P*=0.000017. (F-I) The oxidative stress is increased in *Ercc1^Δ/−^* progeroid mice in comparison to the control mice and is decreased with activin inhibition. Dihydroethidium (DHE) staining highlights the oxidative stress in the left ventricle from control and *Ercc1^Δ/−^* mice. Cardiac frozen sections from untreated control mice (*n*=xx) (F), untreated *Ercc1^Δ/−^* mice (*n*=5) (G), sActRIIB-treated control mice (*n*=4) (H) and sActRIIB-treated *Ercc1^Δ/−^* mice (*n*=6) (I) aged 16 weeks. (J) The intensity of DHE staining was quantified using ImageJ software. One-way ANOVA parametric test was followed by a Tukey post-hoc test to compare the mean from different groups. Results are expressed as mean of intensity±s.d. Control versus *Ercc1^Δ/−^*, *P*=0.014995; control versus control sActRIIB, *P*=0.338970; *Ercc1^Δ/−^* versus control sActRIIB, *P*=0.000672; *Ercc1^Δ/−^* versus *Ercc1^Δ/−^* sActRIIB, *P*=0.038102. *P≤0.05, ***P<0.001. Scale bars: 100 μm.

To determine the extent of accumulated tissue DNA damage, left ventricular tissue sections were stained for γH2AX, which detects DNA double-strand breaks (Mah et al., 2010). γH2AX staining was significantly increased in *Ercc1^Δ/−^* hearts compared with control hearts (Fig. 3A-E) (*P*<0.000001). In *Ercc1^Δ/−^* hearts treated with sActRIIB, there was a decrease in γH2AX staining compared with untreated *Ercc1^Δ/−^* hearts (*P*<0.023025).

There is a close link between the production of reactive oxygen species and subsequent cellular damage in the form of lipid peroxidation, protein oxidation and DNA damage (Cooke et al., 2003; Dizdaroglu and Jaruga, 2012). We determined the extent of oxidative stress using dihydroethidium (DHE) staining as previously described (Wang and Zou, 2018) (Fig. 3F-J). DHE staining increased significantly in *Ercc1^Δ/−^* hearts compared with control hearts (*P*<0.014995) (Fig. 3J). *Ercc1^Δ/−^* mouse hearts had significantly less DHE staining following treatment with sActRIIB (*P*<0.038102) (Fig. 3J).

### Activin blockade induces cardiomyocyte hypertrophy

**Fig. 4.**
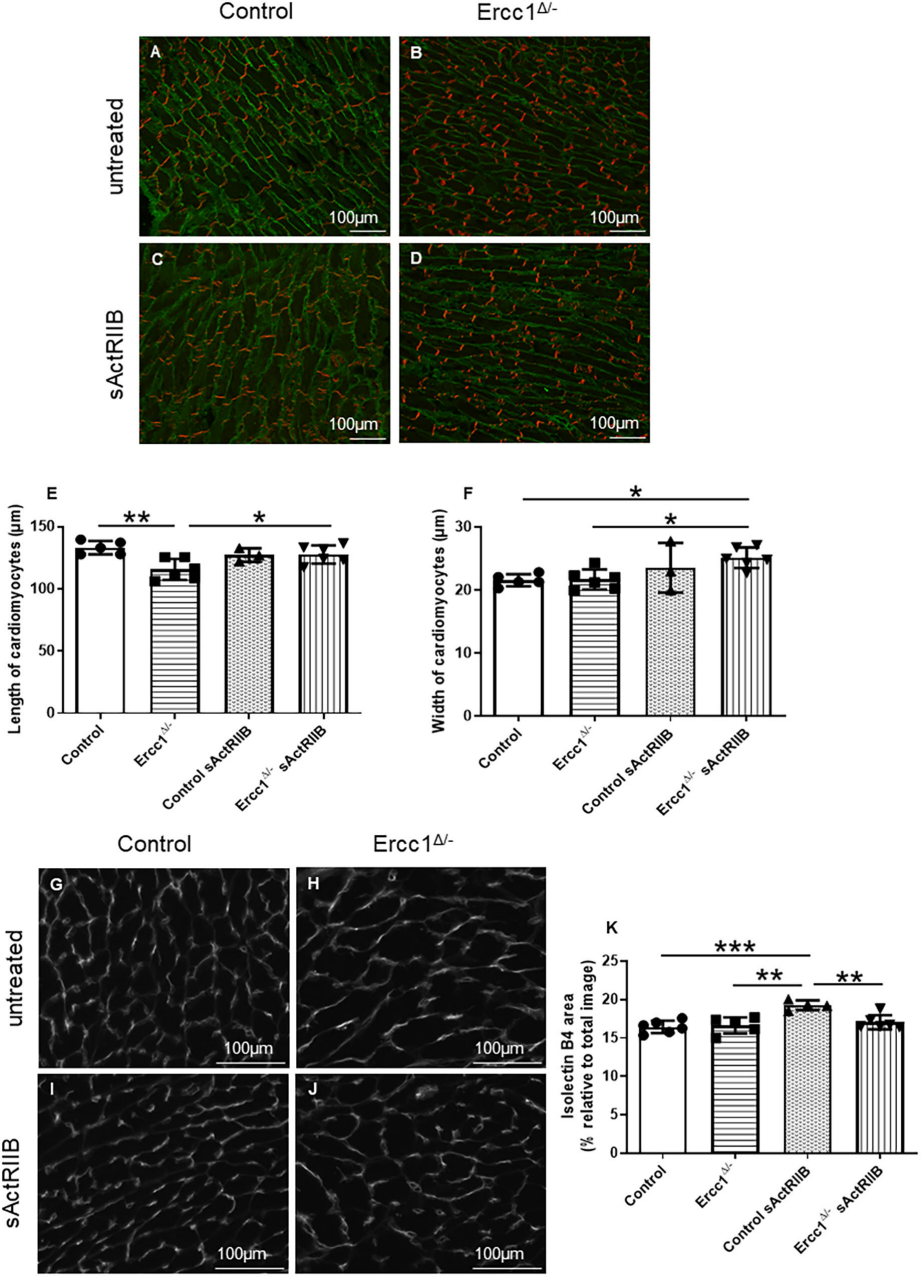
**sActRIIB treatment induces cardiomyocyte hypertrophy in *Ercc1^Δ/−^* mice.** (A-D) Frozen cardiac tissue sections from untreated control mice (*n*=5) (A), untreated *Ercc1^Δ/−^* mice (*n*=6) (B), sActRIIB-treated control mice (*n*=4) (C) and sActRIIB-treated *Ercc1^Δ/−^* mice (*n*=6) (D) aged 16 weeks. (E) The length of cardiomyocytes was quantified using ImageJ software, and then averaged and pooled for each animal. Control versus *Ercc1^Δ/−^*, *P*=0.004042; control versus control sActRIIB, *P*=0.664808; *Ercc1^Δ/−^* versus *Ercc1^Δ/−^* sActRIIB, *P*=0.039810. (F) The width of cardiomyocytes was quantified using ImageJ software, and then averaged and pooled for each animal. One-way ANOVA parametric test was followed by a Tukey post-hoc test to compare the mean from different groups. Results are expressed as mean±s.d. Control versus *Ercc1^Δ/−^*, *P*=0.999609; control versus control sActRIIB, *P*=0.520445; control versus *Ercc1^Δ/−^* sActRIIB, *P*=0.000013; *Ercc1^Δ/−^* versus control sActRIIB, *P*=0.037398; *Ercc1^Δ/−^* versus *Ercc1^Δ/−^* sActRIIB, *P*=0.034548. (G-J) Isolectin B4 staining highlights cardiac vasculature from control and *Ercc1^Δ/−^* mice. Cardiac frozen sections from untreated control mice (*n*=6) (G), untreated *Ercc1^Δ/−^* mice (*n*=5) (H), sActRIIB-treated control mice (*n*=4) (I) and sActRIIB-treated *Ercc1^Δ/−^* mice (*n*=6) (J) aged 16 weeks. (K) Isolectin B4 area was quantified using ImageJ software, and then averaged and pooled for each animal. One-way ANOVA parametric test was followed by a Tukey post-hoc test to compare the mean from different groups. Results are expressed as mean±s.d. Control versus *Ercc1^Δ/−^*, *P*=0.984973; control versus control sActRIIB, *P*=0.000626; *Ercc1^Δ/−^* versus control sActRIIB, *P*=0.001776; *Ercc1^Δ/−^* versus *Ercc1^Δ/−^* sActRIIB, *P*=0.868272; control sActRIIB versus *Ercc1^Δ/−^* sActRIIB, *P*=0.005567. *P≤0.05, **P<0.01, ***P<0.001. Scale bars: 100 μm.

To determine whether the changes to heart weight represented changes to cardiomyocyte size, sections were stained for N-cadherin and wheat germ agglutinin (WGA). N-cadherin allowed longitudinal myocyte length to be determined, and WGA allowed the transverse area to be measured (Fig. 4A-F). The *Ercc1^Δ/−^* mouse hearts displayed a reduced cardiomyocyte length in comparison with control hearts (*P*<0.004042) (Fig. 4E). Moreover, sActRIIB treatment normalised myocyte length in *Ercc1^Δ/−^* hearts (*P*<0.039810) (Fig. 4E). The average myocyte width was unchanged in *Ercc1^Δ/−^* mouse hearts compared with control hearts. However, sActRIIB treatment of Ercc*1^Δ/−^* mice resulted in a significant increase in width compared with untreated *Ercc1^Δ/−^* mice (*P*<0.034548) (Fig. 4F).

To determine whether the microvasculature was altered between the four experimental groups, left ventricular tissue sections were stained using an isolectin B4 antibody, which identifies endothelial cells (Fig. 4G-K). The *Ercc1^Δ/−^* mouse hearts showed no difference in staining compared with control mouse hearts. However, the control mice treated with sActRIIB showed increased staining compared with control mice alone suggesting an increased microvasculature (*P*<0.000626) (Fig. 4K).

### RNA-sequencing analysis for differential gene expression

**Fig. 5.**
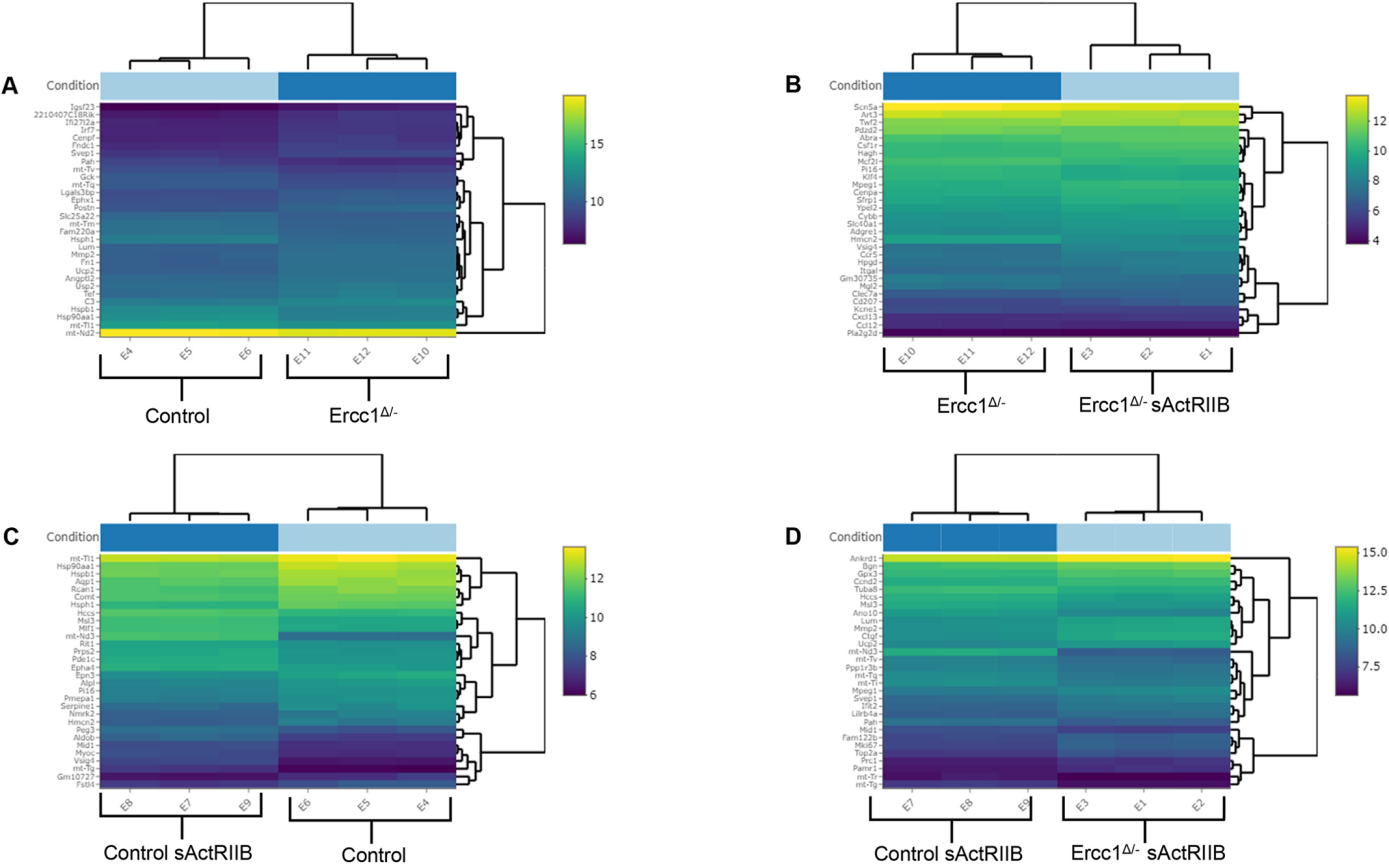
**Heatmap of the top 30 differentially expressed genes sorted by their adjusted *P*-value by plotting their log_2_-transformed expression values in samples.** The bright colours represent the most significantly differentially expressed genes, whereas the dark colour indicates the less significant differentially expressed genes. (A) Pairwise comparison between control mice and *Ercc1^Δ/−^* progeroid mice. (B) Pairwise comparison between untreated *Ercc1^Δ/−^* mice and sActRIIB-treated *Ercc1^Δ/−^* progeroid mice. (C) Pairwise comparison between untreated control mice and sActRIIB-treated control mice. (D) Pairwise comparison between sActRIIB-treated control and sActRIIB-treated *Ercc1^Δ/−^* mice. *n*=3 mice were used for each experimental condition.

To mechanistically understand the impact of the *Ercc1* mutation and how the activin signalling blockade influences cardiac remodelling, RNA-sequencing (RNAseq) analysis was performed on the four experimental groups. The results of this analysis indicated a large number of differentially expressed genes. The heat maps showing the top 30 most significantly altered genes from a pairwise comparison are shown in Fig. 5A-D.

**Fig. 6.**
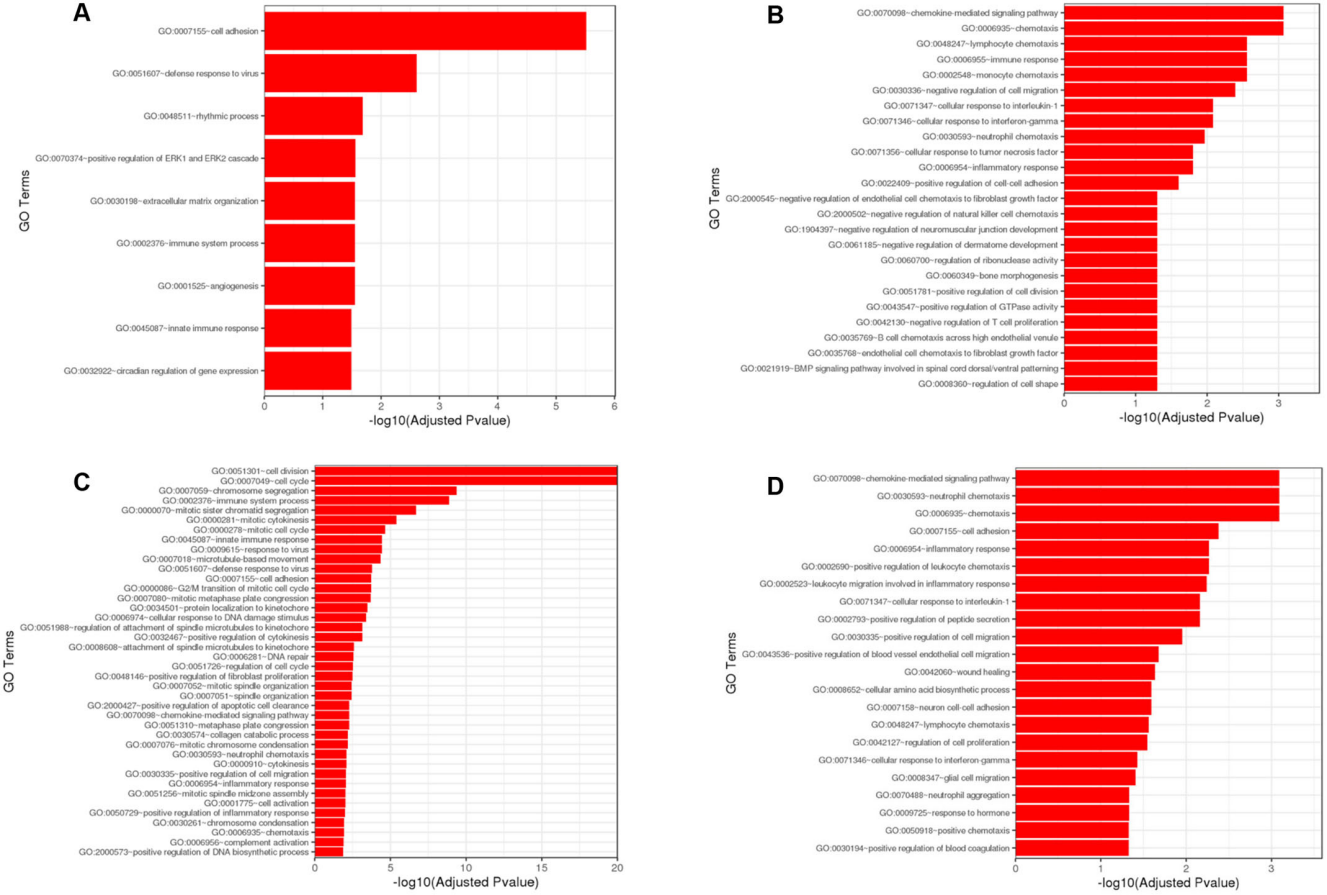
**Gene Ontology (GO) term enrichment analysis from the differentially expressed genes selected with an adjusted *P*-value<0.05 in the differentially expressed gene sets.** Significantly differentially expressed genes were clustered by their gene ontology and the enrichment of gene ontology terms was tested using Fisher exact test (GeneSCF v1.1-p2). (A) Pairwise comparison between control mice and *Ercc1^Δ/−^* mice. (B) Pairwise comparison between untreated *Ercc1^Δ/−^* and treated *Ercc1^Δ/−^* mice. (C) Pairwise comparison between control mice and sActRIIB-treated control mice. (D) Pairwise comparison between sActRIIB-treated control mice and sActRIIB-treated *Ercc1^Δ/−^* mice. *n*=3 mice were used for each experimental condition.

The differentially expressed genes were then clustered by their biological gene ontology to determine which cellular functions had been affected. Genes associated with cell adhesion were the most significantly regulated between the control and *Ercc1^Δ/−^* progeroid mice (Fig. 6A). The chemokine-mediated signalling pathway and chemotaxis were the most significantly altered processes between the untreated and treated *Ercc1^Δ/−^* progeroid mice (Fig. 6B). The chemokine-mediated signalling pathway and chemotaxis were also the most significantly altered processes between the control mice and those treated with sActRIIB (Fig. 6C). Finally, cell division and cell cycle genes were the most significantly altered between the treated control and the treated *Ercc1^Δ/−^* progeroid mice (Fig. 6D).

### Differentially expressed genes relate to experimental phenotype

The differentially expressed genes related to oxidative stress or mitochondrial function and myocyte growth that were significantly different for the two experimental conditions, control versus *Ercc1^Δ/−^* mice, and untreated *Ercc1^Δ/−^* mice versus *Ercc1^Δ/−^* mice treated with sActRIIB, were extracted from the RNAseq analysis (Table 1). The analysis showed that for genes related to oxidative stress, there was an upregulation of leukotriene C4 synthase (*Ltc4s*), and a downregulation of sulfiredoxin 1 (*Srxn1*), uncoupling protein 3 (*Ucp3*) and aquaporin 8 (*Aqp8*) in the *Ercc1^Δ/−^* progeroid mice compared to control mice. For untreated *Ercc1^Δ/−^* mice versus *Ercc1^Δ/−^* mice treated with sActRIIB, there was an upregulation of *Aqp8* and a downregulation of P450 family 2 subfamily E member 1 (*Cyp2e1*) and *Ltc4s* in *Ercc1^Δ/−^* mice treated with sActRIIB. We also examined genes associated with myocyte growth and hypertrophy under the two experimental conditions shown in Table 1. For control versus *Ercc1^Δ/−^* mice, secreted frizzled related protein 2 (*Sfrp2*), myostatin (*Mstn*), SLIT and NTRK like member 4 (*Slitrk4*) and myosin light chain 7 (*Myl7*) were all upregulated, whereas TNF receptor superfamily member 12A (*Tnfrsf12a*) was downregulated in *Ercc1^Δ/−^* mice. For untreated *Ercc1^Δ/−^* mice versus *Ercc1^Δ/−^* mice treated with sActRIIB, fibroblast growth factor 6 (*Fgf6*) was upregulated, whereas WNT inhibitory factor 1 (*Wif1*), *Slitrk4*, *Sfrp2* and *Myl7* were downregulated in *Ercc1^Δ/−^* mice treated with sActRIIB.

**Table 1.**
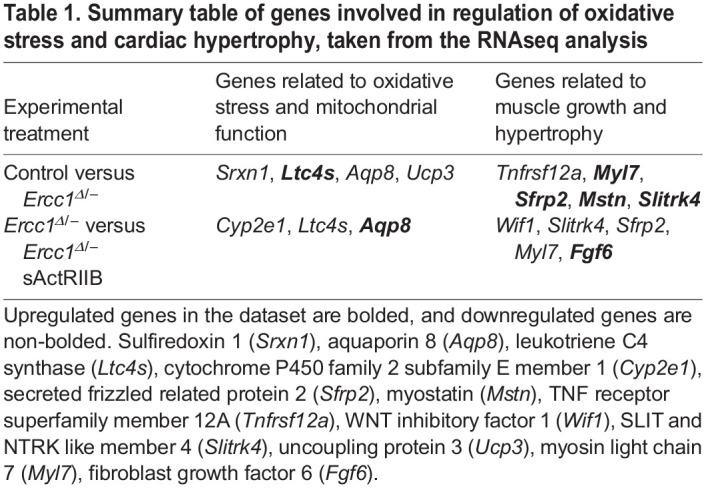
Summary table of genes involved in regulation of oxidative stress and cardiac hypertrophy, taken from the RNAseq analysis

### RNAseq analysis for determining alternatively spliced genes

**Fig. 7.**
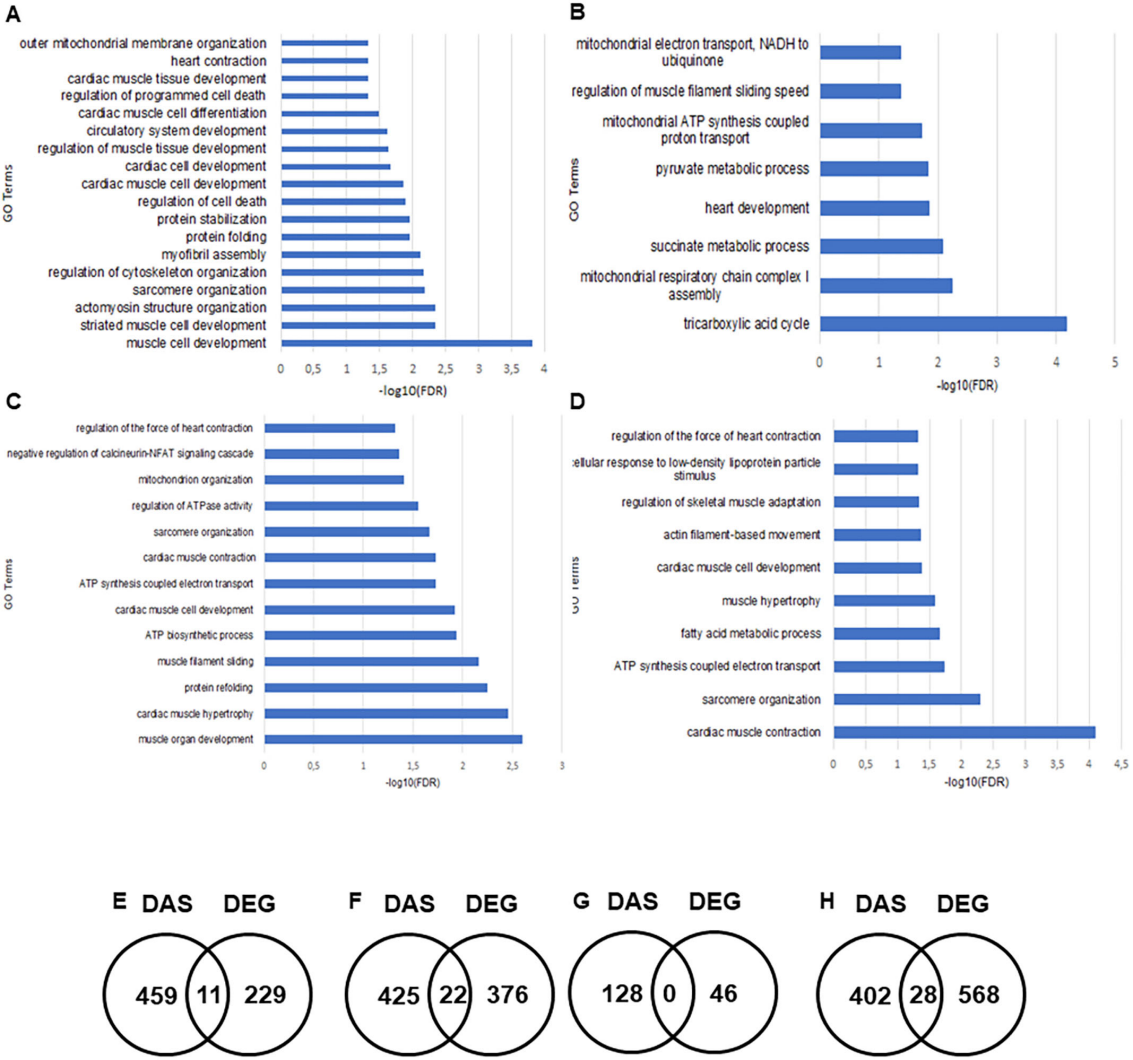
**GO term enrichment analysis from the differentially alternatively spliced genes selected.** Genes were selected from the exon sequencing analysis performed by GENEWIZ^®^. Significantly different alternatively spliced genes were clustered by their gene ontology and the enrichment of gene ontology terms was tested using Fisher exact test with Gene Ontology Resource. *P*-value were corrected with false discovery rate test. (A) Pairwise comparison between control mice and *Ercc1^Δ/−^* mice. (B) Pairwise comparison between *Ercc1^Δ/−^* mice and sActRIIB-treated *Ercc1^Δ/−^* progeroid mice. (C) Pairwise comparison between control mice and sActRIIB-treated control mice. (D) Pairwise comparison between sActRIIB-treated control mice and sActRIIB-treated *Ercc1^Δ/−^* mice. *n*=3 mice were used for each experimental condition. (E-H) Venn diagrams highlight the different and overlapping genes between differentially expressed genes (DEG) and differentially alternatively spliced genes (DAS). Pairwise comparison between control mice and *Ercc1^Δ/−^* mice (E). Pairwise comparison between *Ercc1^Δ/−^* mice and sActRIIB-treated *Ercc1^Δ/−^* progeroid mice (F). Pairwise comparison between control mice and sActRIIB-treated control mice (G). Pairwise comparison between sActRIIB-treated control mice and sActRIIB-treated *Ercc1^Δ/−^* mice (H). *n*=3 mice were used for each experimental condition.

The RNAseq study also highlighted alternatively spliced genes that were differentially expressed from the four experimental groups. These were clustered by their gene ontology using gene ontology resources (Mi et al., 2019). Alternatively spliced genes associated with muscle development and organisation were the most affected between control and the *Ercc1^Δ/−^* mouse hearts (Fig. 7A). Genes related to metabolism were the most significantly affected between the untreated *Ercc1^Δ/−^* mice and *Ercc1^Δ/−^* mice treated with sActRIIB (Fig. 7B). Genes related to muscle development, hypertrophy and sarcomere organisation were the most significantly affected between control mice and those treated with sActRIIB (Fig. 7C). Finally, genes associated with cardiac muscle contraction, sarcomere organisation and hypertrophy were the most affected between the sActRIIB-treated control mice and sActRIIB-treated *Ercc1^Δ/−^* mice (Fig. 7D).

The differentially expressed genes highlighted in the RNAseq study were compared to the differentially expressed alternatively spliced genes to identify those affected by both processes (Fig. 7E-H). The comparison between control and *Ercc1^Δ/−^* mouse hearts indicated 22 overlapping genes (Fig. 7E, Table S1). The pairwise comparison between untreated *Ercc1^Δ/−^* mice and *Ercc1^Δ/−^* mice treated with sActRIIB did not show any overlapping genes (Fig. 7F). The comparison between untreated control mice and control mice treated with sActRIIB showed 11 overlapping genes (Fig. 7G, Table S1), whereas the comparison between control sActRIIB-treated mice and *Ercc1^Δ/−^* sActRIIB-treated mice indicated 28 overlapping genes (Fig. 7H, Table S1).

## DISCUSSION

In this study, we investigated both the impact of *Ercc1* depletion and the blockade of activin signalling on cardiac remodelling and gene expression. This study shows for the first time that *Ercc1^Δ/−^* progeroid mouse hearts displayed significant DNA damage associated with increased oxidative stress. These hearts also had smaller cardiomyocytes and reduced systolic function. Activin blockade significantly reduced DNA damage, oxidative stress and cardiac fibrosis, induced myocyte hypertrophy and improved systolic function in *Ercc1^Δ/−^* hearts. Gene expression analysis from RNAseq identified a large number of differentially expressed and alternatively spliced genes in all groups. The analysis showed that activin blockade decreased pro-oxidant and concomitantly increased antioxidant gene expression. Additionally, activin blockade also decreased the expression of anti-hypertrophic genes and increased the expression of hypertrophic genes. Mechanistically, these changes help to explain the improved morphology and function of *Ercc1^Δ/−^* mouse hearts treated with sActRIIB. Our data also suggest that sActRIIB treatment of *Ercc1^Δ/−^* mice also significantly attenuates cardiac inflammation as a result of decreased cellular damage.

**Fig. 8.**
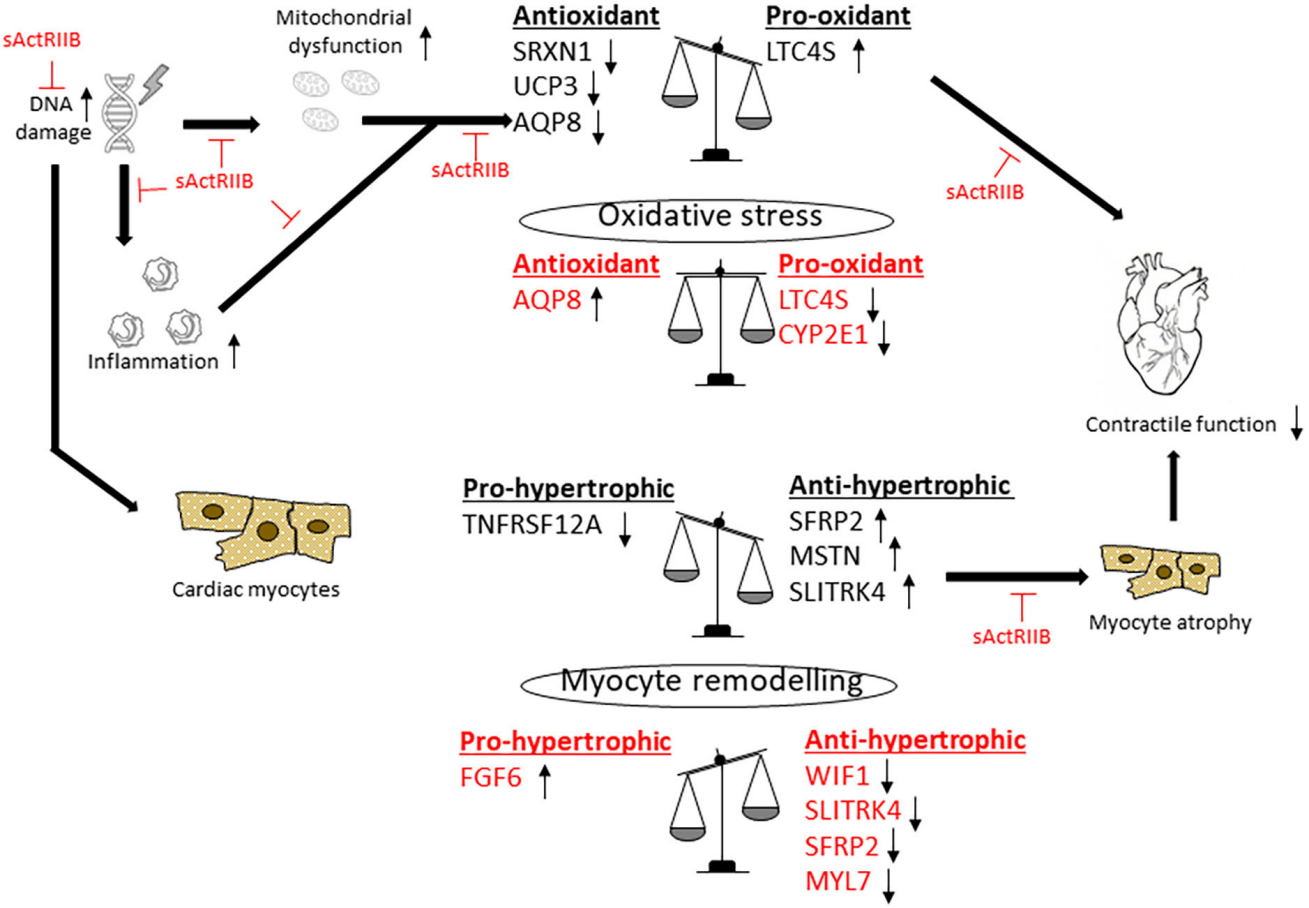
**Diagram showing the proposed mechanism by which the *Ercc1^Δ/−^* mutation induced progeria and inhibition of the activin signalling pathway by sActRIIB-regulated gene expression related to oxidative stress and myocyte remodelling.** In this schematic, genomic DNA damage results in mitochondrial dysfunction and inflammation. This is associated with decreased expression of tissue antioxidants, while increasing pro-oxidant genes, resulting in increased oxidative stress and cardiac dysfunction. DNA damage also results in the upregulation of anti-hypertrophic genes, whilst reducing pro-hypertrophic genes. This results in myocyte atrophy and a reduced contractile function. On the other hand, sActRIIB treatment results in an upregulation of antioxidants, whilst also reducing pro-oxidant genes. In parallel, sActRIIB treatment also shifts the balance in favour of myocyte hypertrophy by decreasing the expression of anti-hypertrophic genes, whilst increasing pro-hypertrophic genes. This increases cardiac systolic function. Therefore, the combined effects of sActRIIB treatment on oxidative stress and myocyte hypertrophy result in improved tissue morphology and contractile function. The genes affected by inhibition of activin signalling via sActRIIB treatment are shown in red.

Oxidative stress is a major source of protein and DNA damage and lipid peroxidation, and is associated with cardiac dysfunction. Our RNAseq analysis showed that the antioxidant genes *Srxn1*, *Ucp3* and *Aqp8* were all downregulated in *Ercc1^Δ/−^* hearts. The upregulation of *Srxn1* has been shown to increase the survival of cardiac progenitor cells and astrocytes by protecting them against oxidative stress (Li et al., 2018; Zhou et al., 2015). *Aqp8* expression is a marker of mitochondrial function (Ikaga et al., 2015), and has been shown to be protective by transporting H_2_O_2_ out of pancreatic cells in the context of diabetes mellitus (Krüger et al., 2021). The downregulation of *Aqp8* in *Ercc1^Δ/−^* hearts would lead to increased oxidative stress and mitochondrial dysfunction. Another antioxidant *Ucp3* that was decreased in *Ercc1^Δ/−^* hearts has been shown to neutralise oxidative stress in myotubes (Barreiro et al., 2009). Taken together, the downregulation of these antioxidants would result in a significant oxidative stress in *Ercc1^Δ/−^* hearts. sActRIIB treatment increased the expression of *Aqp8*, suggesting improved mitochondrial function and reduced oxidative stress as indicated in Fig. 8.

In addition to a decrease in tissue antioxidants, we show increased expression of the pro-oxidant *Ltc4s* in *Ercc1^Δ/−^* hearts. *Ltc4s* has been shown to induce oxidative stress in chemotherapy, resulting in DNA damage (Dvash et al., 2015), so the increase in *Ercc1^Δ/−^* hearts would be associated with inflammation-induced oxidation. Our RNAseq analysis showed that sActRIIB downregulated *Ltc4s*, suggesting reduced inflammation, and another pro-oxidative enzyme *Cyp2e1* in the *Ercc1^Δ/−^* mouse hearts. *Cyp2e1* has been shown to induce oxidative stress in cardiomyocytes, resulting in apoptosis and impaired contractile function (Zhang et al., 2011). Moreover, inhibition of *Cyp2e1* has been shown to improve cardiomyocyte contractility (Ren et al., 2019). The activin signalling pathway has been associated with increased oxidative stress. For example, myostatin was shown to increase oxidative stress through activation of NF-κB which in turn leads to TNF-α secretion (Sriram et al., 2011). Therefore, inhibiting the activin A/myostatin pathway may present a novel means of reducing oxidative stress in the heart. Despite the accumulation of DNA damage and intracellular oxidative stress, *Ercc1^Δ/−^* progeroid mouse hearts did not develop significant fibrosis. Although *Ercc1^Δ/−^* hearts displayed an increase in tissue interstitial space, presumably as a result of cell necrosis and apoptosis, there was no subsequent increase in cardiac fibrosis**.** These findings indicate that *Ercc1^Δ/−^* mice may have functionally impaired cardiac fibroblasts (Robinson et al., 2018). In humans, mutations in the *ERCC1* gene have been shown to affect the activity of fibroblasts (Jaspers et al., 2007), suggesting that DNA damage may directly impair the activity of these cells. In another progeroid model induced by a nuclear lamin A mutation, ECM protein synthesis by fibroblasts was impaired via the Wnt/β-catenin pathways, suggesting that progeria does not always display the hallmarks of normal ageing (Hernandez et al., 2010). Chemokine activity by cardiac macrophages has been shown to lead to impaired fibronectin and collagen deposition. This was associated with impaired remodelling of the ECM through the upregulation of matrix metalloproteinase-2 (*Mmp2*) (Deleon-Pennell et al., 2017).

Our data showed that inhibition of activin signalling resulted in a reduction of cardiac tissue fibrosis and collagen in both *Ercc1^Δ/−^* and control mice. Increased activin signalling is associated with tissue fibrosis as a result of cardiac fibroblast proliferation and differentiation through the activation of the p38-MAPK and ERK1/2 pathway (Hu et al., 2016). Myostatin expression also induced interstitial fibrosis by the activation of the TAK1-MKK3/6 pathway leading to the production of collagen I (Biesemann et al., 2015). This suggests that inhibition of the activin signalling pathway could be used to reduce cardiac fibrosis in ageing, diseased hearts and other conditions that increase fibrosis.

To determine some additional mechanistic aspects in the phenotypes associated with the four treatment groups, we used mRNA sequencing to identify genes that were differentially expressed and spliced. A large number of genes were shown to be significantly targeted in the different treatment groups, and these were grouped by gene ontology. Interestingly, altered expression of a large number of genes suggested a disrupted circadian cycle in *Ercc1^Δ/−^* hearts compared with control hearts. Clock genes such as *Arntl* (*Bmal1*), *Per1*, *Per2*, *Per3*, *Nr1d2*, *Ciart*, *Npas2*, *Nfil3* and *Dbp* were all significantly altered. *Arntl* is expressed out of phase with its physiological repressors, the Per genes: *Per1*, *Per2* and *Per3* (Cao et al., 2021). A human study published by Chen et al. (2016), showed that ageing was associated with a decrease of *Arntl* expression, which was associated with a loss of the rhythmicity of the Per genes. *Arntl* is an important regulator of ageing mechanisms as its activity is vital for cell division and repair. Indeed, the deletion of *Arntl* in mice resulted in a progeroid syndrome with a shorter lifespan, smaller organs, increase of oxidative stress, loss of subcutaneous fat and other ageing-related diseases (Kondratov et al., 2006).

When *Ercc1^Δ/−^* mice were treated with sActRIIB, there were far fewer changes observed in differentially expressed genes. The gene ontology of biological processes suggested that the main gene targets were those associated with the suppression of inflammation. This suggests that the inhibition of activin signalling significantly attenuates tissue inflammation in progeria. The increased inflammation in *Ercc1^Δ/−^* mice is thought to be via activation of the NF-κB signalling pathway (Tilstra et al., 2012). Zhao and colleagues showed that NF-κB activation was associated with increased DNA damage and the activation of DNA damage response pathways (Zhao et al., 2020). Other upregulated chemokines in *Ercc1^Δ/−^* hearts such as *Ccl8*, *Ccl11* and *Ccl12* and secreted factors such as *Retnla* have been shown to be involved in the late phase of inflammation (Yu et al., 2015). *Ccl8* gene expression under the regulation of NF-κB (Halle et al., 2010) was associated with uremic cardiomyopathy (Amador-Martínez et al., 2021). *Ccl11*, or eotaxin, is also known as the protein of senility (Khavinson et al., 2016) and is mainly produced in the heart by the resident fibroblast and the macrophages. Its secretion is required for the eosinophil chemoattraction in myocarditis (Diny et al., 2016). *Ccl11* was also shown to be involved in angiotensin II-induced cardiac hypertrophy through the downstream regulation of pro-hypertrophic genes such as *ANP*, *BNP* and *BMHC* (Zhang et al., 2018).

Our data indicate that the cardiomyocytes from *Ercc1^Δ/−^* hearts were significantly smaller than control hearts and were associated with decreased systolic function. The RNAseq analysis showed that the smaller myocytes were associated with increased expression of the anti-hypertrophic genes *Sfrp2*, *Mstn* and *Sltrk4*. *Sfrp2* inhibits hypertrophy through the Wnt/β-catenin pathway (Wei et al., 2020) whereas MSTN attenuates hypertrophy through its impact on autophagy (Qi et al., 2020). In addition to the increased expression of anti-hypertrophic genes, *Ercc1^Δ/−^* hearts also expressed lower levels of the pro-hypertrophic gene *Tnfrsf12a*. *Tnfrsf12a* has been shown to be an important inducer of cardiac hypertrophy (Yerra et al., 2021). However, the size of the cardiomyocytes relative to heart weight suggests that *Ercc1^Δ/−^* hearts are also likely to have fewer contractile cells. As cardiomyocyte proliferation ceases early in the neonatal period, reduced myocyte number is likely to have occurred during development. Specifically, *Ercc1^Δ/−^* hearts had shorter myocytes whereas myocyte width remained unchanged. Myocyte size is regulated by both preload and afterload and is associated with the addition of sarcomeres in series or parallel, respectively (Opie et al., 2006). Cardiac size can also be affected in conditions that induce cachexia such as cancer and viral infections through the NF-κB pathway (Harvey and Leinwand, 2014). In cancer, the inhibition of NF-κB increased left ventricular mass and function by inducing cardiomyocyte hypertrophy. NF-κB-mediated inflammation also leads to the upregulation of the ubiquitin ligases MuRF1 and atrogin-1 that are known to be involved in myostatin- or activin A-induced skeletal muscle atrophy by increasing protein degradation and inhibiting protein synthesis (Wysong et al., 2011). The activation of MuRF1, atrogin-1 and Foxo3a increase protein degradation through the ubiquitin-proteasome machinery and autophagy (Wang et al., 2015; Ding et al., 2017). MuRF1 and atrogin-1 can also inhibit the canonical cardiac hypertrophic pathway through calcineurin (Razeghi and Taegtmeyer, 2006) and Akt through the downregulation of mTOR signalling pathway (Razeghi and Taegtmeyer, 2005). *Ercc1^Δ/−^* hearts treated with sActRIIB had improved systolic function as measured by increased ejection fraction and fractional shortening. This was associated with increased myocyte hypertrophy. However, stroke volume and cardiac output were not significantly altered, suggesting that changes to filling properties could also be altered by sActRIIB, as shown in a previous study (Rodgers et al., 2009). The RNAseq study highlighted the downregulation of the anti-hypertrophic genes *Wif1*, *Slitrk4*, *Sfrp2* and *Myl7* in the *Ercc1^Δ/−^* mice treated with sActRIIB. *Myl7* has been shown to inhibit cardiac hypertrophy (Sun et al., 2021), whereas *Wif1* impacts hypertrophy through inhibition of the Wnt/β-catenin pathway (Lu et al., 2013). Additionally, sActRIIB treatment of *Ercc1^Δ/−^* mouse hearts increased the expression of *Fgf6*, which is associated with protein synthesis and muscle hypertrophy (Xu et al., 2021). sActRIIB treatment shifts the balance in favour of hypertrophic myocyte growth and, in combination with the reduction in oxidative stress, results in improved systolic function as illustrated in Fig. 8.

Our data showed that although sActRIIB treatment increased the cardiac vasculature in control mouse hearts, it had no impact on *Ercc1^Δ/−^* progeroid hearts. These data indicate that the cellular hypertrophy induced by sActRIIB treatment was not accompanied by significant angiogenesis. The lack of angiogenesis normally associated with myocyte hypertrophy could negate some of the beneficial effects by increasing the diffusion distance for oxygen and nutrients. Durik et al. (2012) showed that *Ercc1^Δ/−^* progeroid mice developed vascular cell senescence, vasodilator dysfunction, vascular stiffness and elevated blood pressure. These vascular changes and the inhibition of angiogenic pathways could explain the impaired response to sActRIIB treatment in *Ercc1^Δ/−^* hearts, despite the increased cardiomyocyte hypertrophy. It is interesting that sActRIIB treatment significantly decreased cardiac size in control hearts, whereas the size of *Ercc1^Δ/−^* hearts remained unchanged. These data are in contrast to skeletal muscle, in which sActRIIB treatment is known to significantly increase muscle mass (Alyodawi et al., 2019). These differences indicate that skeletal and cardiac muscles have differing responses to activin signalling inhibition. Our work here showed that unlike *Ercc1^Δ/−^* mice, treatment of control mice with sActRIIB resulted in a decrease in the weight of the heart. The impact of sActRIIB on cardiac gene expression changes in control mice is clearly evident in heatmap plots (Fig. 5B). Indeed, scrutiny of the gene expression changes through the heatmaps suggests that the impact of sActRIIB was greater in control hearts than in the *Ercc1^Δ/−^* hearts. It is therefore important to understand the cellular origin of these changes in gene expression and their functional consequence for the control mice. The most obvious mediator of these gene changes is the cardiomyocyte population. However, analysis of cardiomyocyte size showed that there were no significant changes induced by sActRIIB treatment of control mice. Therefore, if changes are being induced in cardiomyocytes, they are not affecting their size. It is also worth contemplating the non-contractile component of the heart as a possible reason for the decreased heart weight following treatment with sActRIIB. Conceivable explanations for this point come to light by interrogating the Gene Ontology (GO) term enrichment analysis from the differentially expressed genes, which reveals that a number of activities related to changes in the ECM may have changed, e.g. regulation of protein secretion, changes in cell adhesion molecules and, importantly, regulation of proliferation. Therefore, the changes in these pathways could act on the cardiac fibroblast population resulting in fewer cells, which ultimately produce less ECM as evidenced by our quantification of collagen I (Fig. 2). However, we suggest that the decrease in collagen I levels induced by sActRIIB may not reflect changes in fibrosis (a pathological state) but rather the ECM normally formed to couple structure with function. This seems to be the most plausible source of loss of heart weight. Changes in ECM may also have impacts on myocardial compliance, thereby affecting filling properties during diastole.

Finally, our study shows for the first time differences in gene targets between those that were differentially expressed and alternatively spliced in *Ercc1^Δ/−^* mouse hearts. A small number of genes were both alternatively spliced and expressed in the treatment groups, with the exception of the *Ercc1^Δ/−^* mice that were treated with sActRIIB. This suggests that in *Ercc1^Δ/−^*-induced progeria, the coordination between mRNA synthesis and post-transcriptional regulation is impaired and likely blunts the response to activin-signalling inhibition. Alternative splicing may provide an important means of regulating cellular activities in post-mitotic cells such as cardiomyocytes. In cardiomyopathy, alternative splicing has been shown to target calcium signalling, sarcomere organisation and apoptosis (Hu et al., 2017; Dlamini et al., 2015; Kong et al., 2010). These findings are in keeping with our data indicating that splicing-induced functional changes in proteins are important for regulating cardiac muscle architecture (Kolur et al., 2021; D'Amario et al., 2018), calcium homeostasis (Torac et al., 2014; Wanitchakool et al., 2017), inflammation (Comerford et al., 2006; Wu et al., 2019; Morgan and Harris, 2015) and circadian rhythmicity (Young, 2006; Wang et al., 2021). The numerous changes in response to sActRIIB treatment are likely due to signalling changes downstream of the activin receptor. Studies have shown that inhibition of this pathway has significant impact on both cardiac and skeletal muscle, resulting in both morphological and changes in gene expression (Lodberg, 2021).

In conclusion, we have shown for the first time that a model of attenuated DNA repair is associated with pathological cardiac remodelling and gene expression. However, a significant amount of this phenotype is attenuated by inhibition of the activin signalling pathway using soluble activin receptor treatment. The inhibition of this pathway improved cardiac systolic function, decreased cardiac fibrosis, oxidative stress and DNA damage. Moreover, gene expression analysis showed that inhibition of the activin signalling pathway led to an increase in antioxidants and a decrease in pro-oxidants that explains the decreased oxidative stress. There was also increased expression of pro-hypertrophic and a decrease in anti-hypertrophic genes that was associated with myocyte hypertrophy. We propose that these two mechanisms combine to improve cardiac contractile function following sActRIIB treatment. These data strongly suggest that inhibition of activin signalling may provide a novel means of improving cardiac remodelling that is associated with ageing and pathological stimuli that lead to heart failure.

## MATERIALS AND METHODS

### Ethical approval

All work was done in compliance with the Institutional Ethical Committee. The experiments were performed under a project licence from the United Kingdom Home Office in agreement with the Animals (Scientific Procedures) Act 1986. The University of Reading Animal Care and Ethical Review Committee approved all procedures. Animals were humanely sacrificed via Schedule 1 killing using carbon dioxide.

### *Ercc1^Δ/−^* mouse model and soluble activin treatment

This study was performed mostly on male mice with a hybrid C57BL/6-FVB F1 background (Weeda et al., 1997), except for the RNAseq analyses, for which the untreated *Ercc1^Δ/−^* and *Ercc1^Δ/−^* sActRIIB-treated group contained one female. Transgenic *Ercc1^Δ/−^* and control mice were bred as previously described (Dolle et al., 2011; Weeda et al., 1997). Mice were kept in ventilated cages under specific pathogen-free conditions (20-22°C, 12-12 h light-dark cycle) and fed with food and water *ad libitum*. The *Ercc1^Δ/−^* mice were smaller than the control mice, so the food was given within the cages and water bottles with long nozzles and were used from around 2 weeks of age. Mice were bred and maintained on AIN93G synthetic pellet. The myostatin/activin pathway was inhibited from week 7 to week 16 by intraperitoneal injection (IP) with 10 mg/kg of sActRIIB twice a week. Mice were sacrificed at week 16 as this is the timepoint when death began to occur. Once their body weights were measured with a precision balance, animals were sacrificed, then both tibia were measured with digital callipers, hearts were harvested for the study and the weights were measured with an analytical balance as previously described (Alyodawi et al., 2019). The tissue was stored at −80°C prior to use.

### Heart tissue sectioning

Harvested hearts were embedded in Optimal Cutting Temperature (OCT) medium before being stored at −80°C for at least 24 h. Then transverse sections were collected by cryo-sectioning the OCT blocks at a thickness of 5 µm at −20°C and harvested on slides, before being stored at −80°C.

### Assessing cardiac function by ultrasound

The function of the heart was determined with the Vevo 2100 Ultrasound imager on untreated wild-type mice (*n*=4), untreated *Ercc1^Δ/−^* progeroid mice (*n*=3) and sActRIIB-treated *Ercc1^Δ/−^* progeroid mice (*n*=4) aged 12 weeks and 16 weeks. The cardiac function imaging was performed on M-mode short axis on mice under isoflurane anaesthesia. Prior to starting the cardiac function imaging, the Vevo 2100 software (FUJIFILM Visualsonics) was set up with cardiology and cardiac packages. The image was calibrated for the width at 11.36 mm and depth at 11 mm. To perform the anaesthesia, the mouse was placed in the induction chamber and the oxygen flow was calibrated to 1 l/min and the isoflurane vaporiser to 5%. Once the mouse lost its righting reflex and its breathing pattern became deeper, the isoflurane was decreased to 1.5%. The short axis view M-mode images were analysed by using the Vevo 2100 software in order to measure the cardiac physiological parameters, the left ventricle mass, the stroke volume, the cardiac output, the ejection fraction, the fractional shortening, the left ventricle internal diameter at the end of diastole and the interventricular septum thickness in diastole. The results were presented as a mean of the cardiac function assessed at 12 and 16 weeks of age. There were no age discrepancies between the experimental groups.

### Measuring tissue fibrosis by Picrosirius Red staining

To visualise cardiac fibrosis, sections were stained with Picrosirius Red staining kit (ab150681, Abcam). Sections were fixed in Bouin solution for 15 min at 56°C at room temperature, then washed for 15 min at room temperature in distilled water. Sections were stained in Picrosirius Red for 1 h at room temperature under the fume hood. Slides were differentiated in acidified water, dipped twice. Sections were then washed and dehydrated three times in 100% ethanol before being cleared in xylene for 5 min. Coverslips were mounted with DPX mounting medium. The images were analysed using a bright field microscope (Nikon TE-200) and 10 pictures per animal were captured of the left ventricle area at 400× magnification. The quantification of the Picrosirius Red staining was carried out with ImageJ software 1.53h. The original picture was changed to RGB stack, and only the green channel was kept, and the fibrotic area was measured by the threshold method.

### Interstitial collagen I staining

5 μm-thick cardiac tissue sections were incubated in permeabilization buffer 0.5% Triton-X 100 for 15 min before being washed three times for 5 min in 1× PBS. The slides were incubated for 30 min in blocking buffer containing 5% fetal calf serum before being washed three times for 10 min in 1× PBS. Sections were incubated overnight at 4°C with the rabbit polyclonal anti-collagen I primary antibody (1:200, ab34710, Abcam). Slides were then washed three times for 10 min in 1× PBS before being incubated at room temperature for 1 h with the chicken anti-rabbit Alexa Fluor 488 secondary antibody (1:200). Sections were then washed three times for 5 min. Coverslips were mounted on them with DAPI mounting medium, and slides were kept in the dark at 4°C. The images were analysed using fluorescence microscopy (AxioImager Epifluorescent System) and 10 pictures per animal were taken in the left ventricle area at 400× magnification. The quantification of the collagen I staining was carried out with ImageJ software 1.53h by a threshold method to get the percentage of the area covered by collagen I.

### Measurement of myocyte cell size by WGA and N-cadherin co-staining

5 μm-thick sections were fixed in methanol–acetone (50:50) solution at −20°C for 10 min, then washed. Sections were incubated in permeabilization buffer containing 0.5% Triton-X 100 for 15 min before being washed in 1× PBS. Then, the slides were incubated for 30 min in blocking buffer containing 5% fetal calf serum before being washed in 1× PBS. Sections were incubated overnight at 4°C with the primary antibody anti-N-cadherin (1:200, ab18203, Abcam). Slides were then washed in 1× PBS before being incubated at room temperature for 1 h with the secondary antibody Alexa Fluor 568 (1:200) and the probes of the WGA (1:1000, W11261, Thermo Fisher). Sections were then washed, and coverslips were mounted with DAPI mounting medium. The images were taken using fluorescence microscopy (AxioImager Epifluorescent System) and 10 pictures per animal were captured in the left ventricle area with the double staining at 200× magnification to measure the length and the width of the cardiomyocytes (Figs 3 and 4). For these features, 20 cells were analysed per picture with ImageJ software 1.53h before statistical analysis. The width was measured from the WGA line to WGA line (green), whereas the length was measured from the N-cadherin line to N-cadherin line (red) (intercalated disk). For these features, 20 cells were analysed per image, then each picture was averaged before doing statistical analysis.

### Determination of DNA damage by γH2AX staining

5 μm-thick sections were firstly fixed in 5% paraformaldehyde at room temperature for 15 min, then washed in 1× PBS for 15 min. The sections were then incubated for 15 min in permeabilization buffer containing 0.5% Triton-X 100, washed three times for 5 min in 1× PBS, and incubated for 30 min in blocking buffer containing 5% goat serum. Slides were finally incubated overnight at 4°C with the primary antibody anti- γH2AX (1:200, 9718, Cell Signaling Technology), then washed in 1× PBS before being incubated at room temperature for 1 h with the secondary antibody Alexa Fluor 568 (1:200) (Table 1). Coverslips were mounted with DAPI mounting medium. The images were analysed with fluorescence microscopy (AxioImager Epifluorescent System) and 10 pictures per animal were captured in the left ventricle area with the double staining at 400× magnification to highlight the DNA damage within the nuclei. The analysis was performed with ImageJ software 1.53h. Two counts were performed, one for total number of nuclei and the other for the nuclei positive for γH2AX staining. Finally, for the quantification of DNA damage, we calculated the percentage of positive nuclei.

### Determination of oxidative stress by DHE staining

5 μm-thick sections were first left at room temperature to dry for 15 min, then rehydrated with 1× PBS two times for 2 min. Slides were then incubated with 10 μM DHE (Table 1) for 30 min at 37°C in the dark and then washed with 1× PBS two times for 2 min. Coverslips were mounted with DAPI mounting medium, and slides were kept in the dark at 4°C. The images were analysed using fluorescence microscopy (AxioImager Epifluorescent System) and 10 pictures per animal were captured in the left ventricle area at 200× magnification. The image analysis was done with ImageJ software 1.53h by measuring the intensity of the staining.

### RNAseq and alternative splicing analysis

RNAseq and alternative splicing was performed with 20 mg of the left ventricle from three mice for each experimental condition: control and *Ercc1^Δ/−^* mice, and control and *Ercc1^Δ/−^* mice treated with sActRIIB. RNA extraction, library preparations, sequencing reactions and bioinformatics analyses were performed as a blind study at GENEWIZ^®^, LLC (South Plainfield, NJ, USA).

### Statistical analysis

One-way ANOVA parametric test was used followed by a Tukey post-hoc test to compare the mean of control mice to *Ercc1^Δ/−^* progeroid mice that were untreated and treated with sActRIIB. The results are expressed as mean±s.d. The levels of significance were set at *P≤0.05, **P<0.01 and ***P<0.001. The statistical analysis was performed using GraphPad Prism 9.2.0. The enrichment of gene ontology terms of the differentially expressed genes was tested using Fisher exact test with GeneSCF v1.1-p2 and performed by GENEWIZ^®^. The enrichment of gene ontology terms of the differentially alternative spliced gene was performed with the online tool Gene Ontology Resources. The *P*-value was tested by Fisher exact test and corrected with a false discovery rate test to yield any false negatives.

## Supplementary Material

Supplementary information

## References

[DMM049424C1] Ahmed, M. S., Ikram, S., Bibi, N. and Mir, A. (2018). Hutchinson-gilford progeria syndrome: a premature aging disease. *Mol. Neurobiol.* 55, 4417-4427. 10.1007/s12035-017-0610-7 28660486

[DMM049424C2] Alyodawi, K., Vermeij, W. P., Omairi, S., Kretz, O., Hopkinson, M., Solagna, F., Joch, B., Brandt, R. M. C., Barnhoorn, S., Vliet, N. et al. (2019). Compression of morbidity in a progeroid mouse model through the attenuation of myostatin/activin signalling. *J. Cachexia Sarcopenia Muscle* 10, 662-686. 10.1002/jcsm.12404 30916493 PMC6596402

[DMM049424C3] Amador-Martínez, I., García-Ballhaus, J., Buelna-Chontal, M., Cortés-González, C., Massó, F., Jaisser, F. and Barrera-Chimal, J. (2021). Early inflammatory changes and CC chemokine ligand-8 upregulation in the heart contribute to uremic cardiomyopathy. *FASEB J.* 35, e21761. 10.1096/fj.202100746R 34245616

[DMM049424C4] Ambrosy, A. P., Fonarow, G. C., Butler, J., Chioncel, O., Greene, S. J., Vaduganathan, M., Nodari, S., Lam, C. S. P., Sato, N., Shah, A. N. et al. (2014). The global health and economic burden of hospitalizations for heart failure: lessons learned from hospitalized heart failure registries. *J. Am. Coll. Cardiol.* 63, 1123-1133. 10.1016/j.jacc.2013.11.053 24491689

[DMM049424C5] Arora, H., Chacon, A. H., Choudhary, S., Mcleod, M. P., Meshkov, L., Nouri, K. and Izakovic, J. (2014). Bloom syndrome. *Int. J. Dermatol.* 53, 798-802. 10.1111/ijd.12408 24602044

[DMM049424C6] Barreiro, E., Garcia-Martínez, C., Mas, S., Ametller, E., Gea, J., Argilés, J. M., Busquets, S. and López-Soriano, F. J. (2009). UCP3 overexpression neutralizes oxidative stress rather than nitrosative stress in mouse myotubes. *FEBS Lett.* 583, 350-356. 10.1016/j.febslet.2008.12.023 19101552

[DMM049424C7] Biesemann, N., Mendler, L., Kostin, S., Wietelmann, A., Borchardt, T. and Braun, T. (2015). Myostatin induces interstitial fibrosis in the heart via TAK1 and p38. *Cell Tissue Res.* 361, 779-787. 10.1007/s00441-015-2139-2 25725788

[DMM049424C8] Cao, X., Yang, Y., Selby, C. P., Liu, Z. and Sancar, A. (2021). Molecular mechanism of the repressive phase of the mammalian circadian clock. *Proc. Natl Acad. Sci. USA* 118, e2021174118. 10.1073/pnas.2021174118 33443219 PMC7812753

[DMM049424C9] Chen, C.-Y., Logan, R. W., Ma, T., Lewis, D. A., Tseng, G. C., Sibille, E. and Mcclung, C. A. (2016). Effects of aging on circadian patterns of gene expression in the human prefrontal cortex. *Proc. Natl Acad. Sci. USA* 113, 206. 10.1073/pnas.1508249112 26699485 PMC4711850

[DMM049424C10] Comerford, I., Milasta, S., Morrow, V., Milligan, G. and Nibbs, R. (2006). The chemokine receptor CCX-CKR mediates effective scavenging of CCL19 in vitro. *Eur. J. Immunol.* 36, 1904-1916. 10.1002/eji.200535716 16791897

[DMM049424C11] Cooke, M. S., Evans, M. D., Dizdaroglu, M. and Lunec, J. (2003). Oxidative DNA damage: mechanisms, mutation, and disease. *FASEB J.* 17, 1195-1214. 10.1096/fj.02-0752rev 12832285

[DMM049424C12] Crome, L. and Kanjilal, G. C. (1971). Cockayne's syndrome: case report. *J. Neurol. Neurosurg. Psychiatry* 34, 171-178. 10.1136/jnnp.34.2.171 4999043 PMC493729

[DMM049424C13] D'Amario, D., Gowran, A., Canonico, F., Castiglioni, E., Rovina, D., Santoro, R., Spinelli, P., Adorisio, R., Amodeo, A., Perrucci, G. et al. (2018). Dystrophin cardiomyopathies: clinical management, molecular pathogenesis and evolution towards precision medicine. *J. Clin. Med.* 7, 291. 10.3390/jcm7090291 PMC6162458 30235804

[DMM049424C14] Deleon-Pennell, K. Y., Iyer, R. P., Ero, O. K., Cates, C. A., Flynn, E. R., Cannon, P. L., Jung, M., Shannon, D. A., Garrett, M. R., Buchanan, W. et al. (2017). Periodontal-induced chronic inflammation triggers macrophage secretion of Ccl12 to inhibit fibroblast-mediated cardiac wound healing. *JCI Insight* 2, e94207. 10.1172/jci.insight.94207 PMC5621894 28931761

[DMM049424C15] Ding, H., Zhang, G., Sin, K. W. T., Liu, Z., Lin, R.-K., Li, M. and Li, Y.-P. (2017). Activin A induces skeletal muscle catabolism via p38β mitogen-activated protein kinase. *J. Cachexia Sarcopenia Muscle* 8, 202-212. 10.1002/jcsm.12145 27897407 PMC5377410

[DMM049424C16] Diny, N. L., Hou, X., Barin, J. G., Chen, G., Talor, M. V., Schaub, J., Russell, S. D., Klingel, K., Rose, N. R. and Čiháková, D. (2016). Macrophages and cardiac fibroblasts are the main producers of eotaxins and regulate eosinophil trafficking to the heart. *Eur. J. Immunol.* 46, 2749-2760. 10.1002/eji.201646557 27621211 PMC5404278

[DMM049424C17] Dizdaroglu, M. and Jaruga, P. (2012). Mechanisms of free radical-induced damage to DNA. *Free Radic. Res.* 46, 382-419. 10.3109/10715762.2011.653969 22276778

[DMM049424C18] Dlamini, Z., Tshidino, S. C. and Hull, R. (2015). Abnormalities in alternative splicing of apoptotic genes and cardiovascular diseases. *Int. J. Mol. Sci.* 16, 27171-27190. 10.3390/ijms161126017 26580598 PMC4661875

[DMM049424C19] Dollé, M. E., Kuiper, R. V., Roodbergen, M., Robinson, J., De Vlugt, S., Wijnhoven, S. W. P., Beems, R. B., De La Fonteyne, L., De With, P., Van Der Pluijm, I. et al. (2011). Broad segmental progeroid changes in short-lived Ercc1(−/Δ7) mice. *Pathobiol Aging Age Relat. Dis.* 1, 7219. 10.3402/pba.v1i0.7219 PMC3417667 22953029

[DMM049424C20] Durik, M., Kavousi, M., Van Der Pluijm, I., Isaacs, A., Cheng, C., Verdonk, K., Loot, A. E., Oeseburg, H., Bhaggoe, U. M., Leijten, F. et al. (2012). Nucleotide excision DNA repair is associated with age-related vascular dysfunction. *Circulation* 126, 468-478. 10.1161/CIRCULATIONAHA.112.104380 22705887 PMC3430727

[DMM049424C21] Dvash, E., Har-Tal, M., Barak, S., Meir, O. and Rubinstein, M. (2015). Leukotriene C4 is the major trigger of stress-induced oxidative DNA damage. *Nat. Commun.* 6, 10112. 10.1038/ncomms10112 26656251 PMC4682057

[DMM049424C22] Gurkar, A. U. and Niedernhofer, L. J. (2015). Comparison of mice with accelerated aging caused by distinct mechanisms. *Exp. Gerontol.* 68, 43-50. 10.1016/j.exger.2015.01.045 25617508 PMC4464936

[DMM049424C23] Halle, M., Gabrielsen, A., Paulsson-Berne, G., Gahm, C., Agardh, H. E., Farnebo, F. and Tornvall, P. (2010). Sustained inflammation due to nuclear factor-kappa B activation in irradiated human arteries. *J. Am. Coll. Cardiol.* 55, 1227-1236. 10.1016/j.jacc.2009.10.047 20298930

[DMM049424C24] Hansson, L. (1998). Hypertension-induced congestive heart failure. *Scand. Cardiovasc. J.* 47, 5-7. 10.1080/140174398427974 9540127

[DMM049424C25] Harvey, P. A. and Leinwand, L. A. (2014). Chapter 3 - cardiac atrophy and remodeling. In *Cellular and Molecular Pathobiology of Cardiovascular Disease* (ed. M.S. Willis, J.W. Homeister and J.R. Stone), pp. 37-50. San Diego: Academic Press.

[DMM049424C26] Hernandez, L., Roux, K. J., Wong, E. S. M., Mounkes, L. C., Mutalif, R., Navasankari, R., Rai, B., Cool, S., Jeong, J.-W., Wang, H. et al. (2010). Functional coupling between the extracellular matrix and nuclear lamina by Wnt signaling in progeria. *Dev. Cell* 19, 413-425. 10.1016/j.devcel.2010.08.013 20833363 PMC2953243

[DMM049424C27] Hu, J., Wang, X., Wei, S.-M., Tang, Y.-H., Zhou, Q. and Huang, C.-X. (2016). Activin A stimulates the proliferation and differentiation of cardiac fibroblasts via the ERK1/2 and p38-MAPK pathways. *Eur. J. Pharmacol.* 789, 319-327. 10.1016/j.ejphar.2016.07.053 27477354

[DMM049424C28] Hu, Z., Liang, M. C. and Soong, T. W. (2017). Alternative splicing of L-type Ca(V)1.2 calcium channels: implications in cardiovascular diseases. *Genes (Basel)* 8, 344. 10.3390/genes8120344 PMC5748662 29186814

[DMM049424C29] Ikaga, R., Namekata, I., Kotiadis, V. N., Ogawa, H., Duchen, M. R., Tanaka, H. and Iida-Tanaka, N. (2015). Knockdown of aquaporin-8 induces mitochondrial dysfunction in 3T3-L1 cells. *Biochem. Biophys. Rep.* 4, 187-195. 10.1016/j.bbrep.2015.09.009 29124204 PMC5668916

[DMM049424C30] Jaspers, N. G. J., Raams, A., Silengo, M. C., Wijgers, N., Niedernhofer, L. J., Robinson, A. R., Giglia-Mari, G., Hoogstraten, D., Kleijer, W. J., Hoeijmakers, J. H. J. et al. (2007). First reported patient with human ERCC1 deficiency has cerebro-oculo-facio-skeletal syndrome with a mild defect in nucleotide excision repair and severe developmental failure. *Am. J. Hum. Genet.* 80, 457-466. 10.1086/512486 17273966 PMC1821117

[DMM049424C31] Jenča, D., Melenovský, V., Stehlik, J., Staně, V., Kettner, J., Kautzner, J., Adámková, V. and Wohlfahrt, P. (2021). Heart failure after myocardial infarction: incidence and predictors. *ESC Heart Failure* 8, 222-237. 10.1002/ehf2.13144 33319509 PMC7835562

[DMM049424C32] Karikkineth, A. C., Scheibye-Knudsen, M., Fivenson, E., Croteau, D. L. and Bohr, V. A. (2017). Cockayne syndrome: clinical features, model systems and pathways. *Ageing Res. Rev.* 33, 3-17. 10.1016/j.arr.2016.08.002 27507608 PMC5195851

[DMM049424C33] Kashiyama, K., Nakazawa, Y., Pilz, D. T., Guo, C., Shimada, M., Sasaki, K., Fawcett, H., Wing, J. F., Lewin, S. O., Carr, L. et al. (2013). Malfunction of nuclease ERCC1-XPF results in diverse clinical manifestations and causes Cockayne syndrome, xeroderma pigmentosum, and Fanconi anemia. *Am. J. Hum. Genet.* 92, 807-819. 10.1016/j.ajhg.2013.04.007 23623389 PMC3644632

[DMM049424C34] Kenchaiah, S., Evans, J. C., Levy, D., Wilson, P. W. F., Benjamin, E. J., Larson, M. G., Kannel, W. B. and Vasan, R. S. (2002). Obesity and the risk of heart failure. *N. Engl. J. Med.* 347, 305-313. 10.1056/NEJMoa020245 12151467

[DMM049424C35] Khavinson, V. K., Kuznik, B. I., Ryzhak, G. A., Linkova, N. S., Kozina, L. S. and Sall, T. S. (2016). [“Protein of senility” CCL11, “protein of juvenility” GDF11 and their role in age-related pathology]. *Adv. Gerontol.* 29, 722-731. 28556640

[DMM049424C36] Kolur, V., Vastrad, B., Vastrad, C., Kotturshetti, S. and Tengli, A. (2021). Identification of candidate biomarkers and therapeutic agents for heart failure by bioinformatics analysis. *BMC Cardiovasc. Disord.* 21, 329. 10.1186/s12872-021-02146-8 34218797 PMC8256614

[DMM049424C37] Kondratov, R. V., Kondratova, A. A., Gorbacheva, V. Y., Vykhovanets, O. V. and Antoch, M. P. (2006). Early aging and age-related pathologies in mice deficient in BMAL1, the core componentof the circadian clock. *Genes Dev.* 20, 1868-1873. 10.1101/gad.1432206 16847346 PMC1522083

[DMM049424C38] Kong, S. W., Hu, Y. W., Ho, J. W. K., Ikeda, S., Polster, S., John, R., Hall, J. L., Bisping, E., Pieske, B., Dos Remedios, C. G. et al. (2010). Heart failure-associated changes in RNA splicing of sarcomere genes. *Circ. Cardiovasc Genet.* 3, 138-146. 10.1161/CIRCGENETICS.109.904698 20124440 PMC3073230

[DMM049424C39] Krüger, C., Waldeck-Weiermair, M., Kaynert, J., Pokrant, T., Komaragiri, Y., Otto, O., Michel, T. and Elsner, M. (2021). AQP8 is a crucial H(2)O(2) transporter in insulin-producing RINm5F cells. *Redox Biol.* 43, 101962. 10.1016/j.redox.2021.101962 33892285 PMC8082690

[DMM049424C40] Lazzeroni, D., Rimoldi, O. and Camici, P. G. (2016). From left ventricular hypertrophy to dysfunction and failure. *Circ. J.* 80, 555-564. 10.1253/circj.CJ-16-0062 26853555

[DMM049424C41] Lee, S. J., Reed, L. A., Davies, M. V., Girgenrath, S., Goad, M. E. P., Tomkinson, K. N., Wright, J. F., Barker, C., Ehrmantraut, G., Holmstrom, J. et al. (2005). Regulation of muscle growth by multiple ligands signaling through activin type II receptors. *Proc. Natl. Acad. Sci. USA* 102, 18117-18122. 10.1073/pnas.0505996102 16330774 PMC1306793

[DMM049424C42] Li, X., He, P., Wang, X.-L., Zhang, S., Devejian, N., Bennett, E. and Cai, C. (2018). Sulfiredoxin-1 enhances cardiac progenitor cell survival against oxidative stress via the upregulation of the ERK/NRF2 signal pathway. *Free Radic. Biol. Med.* 123, 8-19. 10.1016/j.freeradbiomed.2018.05.060 29772252 PMC5999586

[DMM049424C43] Licher, S., Darweesh, S. K. L., Wolters, F. J., Fani, L., Heshmatollah, A., Mutlu, U., Koudstaal, P. J., Heeringa, J., Leening, M. J. G., Ikram, M. K. et al. (2019). Lifetime risk of common neurological diseases in the elderly population. *J. Neurol. Neurosurg. Psychiatry* 90, 148-156. 10.1136/jnnp-2018-318650 30279211

[DMM049424C44] Lodberg, A. (2021). Principles of the activin receptor signaling pathway and its inhibition. *Cytokine Growth Factor. Rev.* 60, 1-17. 10.1016/j.cytogfr.2021.04.001 33933900

[DMM049424C45] López-Otín, C., Blasco, M. A., Partridge, L., Serrano, M. and Kroemer, G. (2013). The hallmarks of aging. *Cell* 153, 1194-1217. 10.1016/j.cell.2013.05.039 23746838 PMC3836174

[DMM049424C46] Loumaye, A., De Barsy, M., Nachit, M., Lause, P., Frateur, L., Van Maanen, A., Trefois, P., Gruson, D. and Thissen, J.-P. (2015). Role of Activin A and myostatin in human cancer cachexia. *J. Clin. Endocrinol. Metab.* 100, 2030-2038. 10.1210/jc.2014-4318 25751105

[DMM049424C47] Lu, D., Dong, W., Zhang, X., Quan, X., Bao, D., Lu, Y. and Zhang, L. (2013). WIF1 causes dysfunction of heart in transgenic mice. *Transgenic Res.* 22, 1179-1189. 10.1007/s11248-013-9738-z 23921644 PMC3835953

[DMM049424C48] Mah, L.-J., El-Osta, A. and Karagiannis, T. C. (2010). γH2AX: a sensitive molecular marker of DNA damage and repair. *Leukemia* 24, 679-686. 10.1038/leu.2010.6 20130602

[DMM049424C49] Massip, L., Garand, C., Turaga, R. V. N., Deschênes, F., Thorin, E. and Lebel, M. (2006). Increased insulin, triglycerides, reactive oxygen species, and cardiac fibrosis in mice with a mutation in the helicase domain of the Werner syndrome gene homologue. *Exp. Gerontol.* 41, 157-168. 10.1016/j.exger.2005.10.011 16330174

[DMM049424C50] Mi, H., Muruganujan, A., Ebert, D., Huang, X. and Thomas, P. D. (2019). PANTHER version 14: more genomes, a new PANTHER GO-slim and improvements in enrichment analysis tools. *Nucleic Acids Res.* 47, D419-D426. 10.1093/nar/gky1038 30407594 PMC6323939

[DMM049424C51] Morgan, B. P. and Harris, C. L. (2015). Complement, a target for therapy in inflammatory and degenerative diseases. *Nat. Rev Drug Discov.* 14, 857-877. 10.1038/nrd4657 26493766 PMC7098197

[DMM049424C52] Murphy, S. P., Kakkar, R., McCarthy, C. P. and Januzzi, J. L. (2020). Inflammation in Heart Failure: JACC State-of-the-Art Review. *J. Am. Coll. Cardiol.* 75, 1324-1340. 10.1016/j.jacc.2020.01.014 32192660

[DMM049424C53] Niccoli, T. and Partridge, L. (2012). Ageing as a risk factor for disease. *Curr. Biol.* 22, R741-R752. 10.1016/j.cub.2012.07.024 22975005

[DMM049424C54] Niedernhofer, L. J., Garinis, G. A., Raams, A., Lalai, A. S., Robinson, A. R., Appeldoorn, E., Odijk, H., Oostendorp, R., Ahmad, A., Van Leeuwen, W. et al. (2006). A new progeroid syndrome reveals that genotoxic stress suppresses the somatotroph axis. *Nature* 444, 1038-1043. 10.1038/nature05456 17183314

[DMM049424C55] North, B. J. and Sinclair, D. A. (2012). The intersection between aging and cardiovascular disease. *Circ. Res.* 110, 1097-1108. 10.1161/CIRCRESAHA.111.246876 22499900 PMC3366686

[DMM049424C56] Olive, M., Harten, I., Mitchell, R., Beers, J. K., Djabali, K., Cao, K., Erdos, M. R., Blair, C., Funke, B., Smoot, L. et al. (2010). Cardiovascular pathology in Hutchinson-Gilford progeria: correlation with the vascular pathology of aging. *Arterioscler. Thromb. Vasc. Biol.* 30, 2301-2309. 10.1161/ATVBAHA.110.209460 20798379 PMC2965471

[DMM049424C57] Opie, L. H., Commerford, P. J., Gersh, B. J. and Pfeffer, M. A. (2006). Controversies in ventricular remodelling. *The Lancet* 367, 356-367. 10.1016/S0140-6736(06)68074-4 16443044

[DMM049424C58] Oshima, J., Martin, G. M. and Hisama, F. M. (1993). Werner syndrome. In *GeneReviews(^®^)* (ed. M. P. Adam et al.). Seattle: University of Washington. 20301687

[DMM049424C59] Qi, H., Ren, J., Ba, L., Song, C., Zhang, Q., Cao, Y., Shi, P., Fu, B., Liu, Y. and Sun, H. (2020). MSTN attenuates cardiac hypertrophy through inhibition of excessive cardiac autophagy by blocking AMPK /mTOR and miR-128/PPARγ/NF-κB. *Mol. Ther. Nucleic Acids* 19, 507-522. 10.1016/j.omtn.2019.12.003 31923740 PMC6951838

[DMM049424C60] Querejeta, R., López, B., González, A., Sánchez, E., Larman, M., Martínez Ubago, J. L. and Díez, J. (2004). Increased collagen type I synthesis in patients with heart failure of hypertensive origin. *Circulation* 110, 1263-1268. 10.1161/01.CIR.0000140973.60992.9A 15313958

[DMM049424C61] Razeghi, P. and Taegtmeyer, H. (2005). Cardiac remodeling. *Circ. Res.* 97, 964-966. 10.1161/01.RES.0000193563.53859.3e 16284188

[DMM049424C62] Razeghi, P. and Taegtmeyer, H. (2006). Hypertrophy and atrophy of the heart: the other side of remodeling. *Ann. N. Y. Acad. Sci.* 1080, 110-119. 10.1196/annals.1380.011 17132779

[DMM049424C63] Ren, J., Pei, Z., Chen, X., Berg, M. J., Matrougui, K., Zhang, Q.-h. and Zhang, Y. (2019). Inhibition of CYP2E1 attenuates myocardial dysfunction in a murine model of insulin resistance through NLRP3-mediated regulation of mitophagy. *Biochim. Biophys. Acta. Mol. Basis Dis.* 1865, 206-217. 10.1016/j.bbadis.2018.08.017 30463689

[DMM049424C64] Robinson, A. R., Yousefzadeh, M. J., Rozgaja, T. A., Wang, J., Li, X., Tilstra, J. S., Feldman, C. H., Gregg, S. Q., Johnson, C. H., Skoda, E. M. et al. (2018). Spontaneous DNA damage to the nuclear genome promotes senescence, redox imbalance and aging. *Redox Biol.* 17, 259-273. 10.1016/j.redox.2018.04.007 29747066 PMC6006678

[DMM049424C65] Rodgers, B. D., Interlichia, J. P., Garikipati, D. K., Mamidi, R., Chandra, M., Nelson, O. L., Murry, C. E. and Santana, L. F. (2009). Myostatin represses physiological hypertrophy of the heart and excitation-contraction coupling. *J. Physiol.* 587, 4873-4886. 10.1113/jphysiol.2009.172544 19736304 PMC2770153

[DMM049424C66] Roh, J. D., Hobson, R., Chaudhari, V., Quintero, P., Yeri, A., Benson, M., Xiao, C., Zlotoff, D., Bezzerides, V., Houstis, N. et al. (2019). Activin type II receptor signaling in cardiac aging and heart failure. *Sci. Transl. Med.* 11, eaau8680. 10.1126/scitranslmed.aau8680 30842316 PMC7124007

[DMM049424C67] Sepe, S., Milanese, C., Gabriels, S., Derks, K. W. J., Payan-Gomez, C., Van Ijcken, W. F. J., Rijksen, Y. M. A., Nigg, A. L., Moreno, S., Cerri, S. et al. (2016). Inefficient DNA repair is an aging-related modifier of Parkinson's disease. *Cell Rep.* 15, 1866-1875. 10.1016/j.celrep.2016.04.071 27210754 PMC4893155

[DMM049424C68] Sharma, K., Darvas, M., Keene, C. D., Niedernhofer, L. J. and Ladiges, W. (2018). Modeling Alzheimer's disease in progeria mice. An age-related concept. *Pathobiol. Aging. Age Relat Dis.* 8, 1524815. 10.1080/20010001.2018.1524815 30319737 PMC6179061

[DMM049424C69] Sriram, S., Subramanian, S., Sathiakumar, D., Venkatesh, R., Salerno, M. S., Mcfarlane, C. D., Kambadur, R. and Sharma, M. (2011). Modulation of reactive oxygen species in skeletal muscle by myostatin is mediated through NF-κB. *Aging Cell* 10, 931-948. 10.1111/j.1474-9726.2011.00734.x 21771249 PMC5028794

[DMM049424C70] Sun, J., Guo, X., Yu, P., Liang, J., Mo, Z., Zhang, M., Yang, L., Huang, X., Hu, B., Liu, J. et al. (2021). Vasorin deficiency leads to cardiac hypertrophy by targeting MYL7 in young mice. *J. Cell. Mol. Med.* 26, 88-98. 10.1111/jcmm.17034 34854218 PMC8742182

[DMM049424C71] Tilstra, J. S., Robinson, A. R., Wang, J., Gregg, S. Q., Clauson, C. L., Reay, D. P., Nasto, L. A., St Croix, C. M., Usas, A., Vo, N. et al. (2012). NF-κB inhibition delays DNA damage-induced senescence and aging in mice. *J. Clin. Invest.* 122, 2601-2612. 10.1172/JCI45785 22706308 PMC3386805

[DMM049424C72] Torac, E., Gaman, L. and Atanasiu, V. (2014). The regulator of calcineurin (RCAN1) an important factor involved in atherosclerosis and cardiovascular diseases development. *J. Med. Life* 7, 481-487. 25713607 PMC4316123

[DMM049424C73] Tune, J. D., Goodwill, A. G., Sassoon, D. J. and Mather, K. J. (2017). Cardiovascular consequences of metabolic syndrome. *Transl. Res.* 183, 57-70. 10.1016/j.trsl.2017.01.001 28130064 PMC5393930

[DMM049424C74] Wang, D.-T., Yang, Y.-J., Huang, R.-H., Zhang, Z.-H. and Lin, X. (2015). Myostatin activates the ubiquitin-proteasome and autophagy-lysosome systems contributing to muscle wasting in chronic kidney disease. *Oxid. Med. Cell. Longev.* 2015, 684965. 10.1155/2015/684965 26448817 PMC4584061

[DMM049424C75] Wang, Q. and Zou, M.-H. (2018). Measurement of reactive oxygen species (ROS) and mitochondrial ROS in AMPK knockout mice blood vessels. *Method. Mol. Biol. (Clifton, N.J.)* 1732, 507-517. 10.1007/978-1-4939-7598-3_32 PMC6407612 29480496

[DMM049424C76] Wang, Y., Jiang, W., Chen, H., Zhou, H., Liu, Z., Liu, Z., Liu, Z., Zhou, Y., Zhou, X., Yu, L. et al. (2021). Sympathetic nervous system mediates cardiac remodeling after myocardial infarction in a circadian disruption model. *Front. Cardiovasc. Med.* 8, 212. 10.3389/fcvm.2021.668387 PMC8032890 33842566

[DMM049424C77] Wanitchakool, P., Ousingsawat, J., Sirianant, L., Cabrita, I., Faria, D., Schreiber, R. and Kunzelmann, K. (2017). Cellular defects by deletion of ANO10 are due to deregulated local calcium signaling. *Cell. Signal.* 30, 41-49. 10.1016/j.cellsig.2016.11.006 27838374

[DMM049424C78] Weeda, G., Donker, I., De Wit, J., Morreau, H., Janssens, R., Vissers, C. J., Nigg, A., Van Steeg, H., Bootsma, D. and Hoeijmakers, J. H. J. (1997). Disruption of mouse ERCC1 results in a novel repair syndrome with growth failure, nuclear abnormalities and senescence. *Curr. Biol.* 7, 427-439. 10.1016/S0960-9822(06)00190-4 9197240

[DMM049424C79] Wei, W. Y., Zhao, Q., Zhang, W.-z., Wang, M.-j., Li, Y., Wang, S.-z. and Zhang, N. (2020). Secreted frizzled-related protein 2 prevents pressure-overload-induced cardiac hypertrophy by targeting the Wnt/β-catenin pathway. *Mol. Cell. Biochem.* 472, 241-251. 10.1007/s11010-020-03802-x 32632611 PMC7338134

[DMM049424C80] Wu, M., Skaug, B., Bi, X., Mills, T., Salazar, G., Zhou, X., Reveille, J., Agarwal, S. K., Blackburn, M. R., Mayes, M. D. et al. (2019). Interferon regulatory factor 7 (IRF7) represents a link between inflammation and fibrosis in the pathogenesis of systemic sclerosis. *Ann. Rheum. Dis.* 78, 1583-1591. 10.1136/annrheumdis-2019-215208 31439591 PMC7167109

[DMM049424C81] Wysong, A., Couch, M., Shadfar, S., Li, L., Rodriguez, J. E., Asher, S., Yin, X., Gore, M., Baldwin, A., Patterson, C. et al. (2011). NF-κB inhibition protects against tumor-induced cardiac atrophy in vivo. *Am. J. Pathol.* 178, 1059-1068. 10.1016/j.ajpath.2010.12.009 21356358 PMC3070568

[DMM049424C82] Xu, B., Liu, C., Zhang, H., Zhang, R., Tang, M., Huang, Y., Jin, L., Xu, L., Hu, C. and Jia, W. (2021). Skeletal muscle-targeted delivery of Fgf6 protects mice from diet-induced obesity and insulin resistance. *JCI Insight* 6, e149969. 10.1172/jci.insight.149969 34491915 PMC8525645

[DMM049424C83] Yerra, V. G., Batchu, S. N., Kabir, G., Advani, S. L., Liu, Y., Siddiqi, F. S., Connelly, K. A. and Advani, A. (2021). Empagliflozin disrupts a Tnfrsf12a-mediated feed forward loop that promotes left ventricular hypertrophy. *Cardiovasc. Drugs Ther*. 10.1007/s10557-021-07190-2 33886003

[DMM049424C84] Yin, F. C., Spurgeon, H. A., Rakusan, K., Weisfeldt, M. L. and Lakatta, E. G. (1982). Use of tibial length to quantify cardiac hypertrophy: application in the aging rat. *Am. J. Physiol.* 243, H941-H947. 10.1152/ajpheart.1982.243.6.H941 6216817

[DMM049424C85] Yndestad, A., Ueland, T., Øie, E., Florholmen, G., Halvorsen, B., Attramadal, H., Simonsen, S., Frøland, S. S., Gullestad, L., Christensen, G. et al. (2004). Elevated Levels of Activin A in Heart Failure. *Circulation* 109, 1379-1385. 10.1161/01.CIR.0000120704.97934.41 14993131

[DMM049424C86] Young, M. E. (2006). The circadian clock within the heart: potential influence on myocardial gene expression, metabolism, and function. *Am. J. Physiol. Heart Circ. Physiol.* 290, H1-H16. 10.1152/ajpheart.00582.2005 16373589

[DMM049424C87] Yu, L., Wu, X., Wei, J., Liao, Q., Xu, L., Luo, S., Zeng, X., Zhao, Y., Lv, Z. and Wu, Z. (2015). Preliminary expression profile of cytokines in brain tissue of BALB/c mice with Angiostrongylus cantonensis infection. *Parasit. Vectors* 8, 328. 10.1186/s13071-015-0939-6 26070790 PMC4476182

[DMM049424C88] Yuen, M. K., Chandra Rodrigo, M. R., Law Min, J. C. and Antonio Tong, C. K. (2001). Myocardial ischemia and delayed recovery after anesthesia in a patient with Cockayne syndrome: a case report. *J. Oral Maxillofac. Surg.* 59, 1488-1491. 10.1053/joms.2001.28291 11732042

[DMM049424C89] Zhang, W., Lu, D., Dong, W., Zhang, L., Zhang, X., Quan, X., Ma, C., Lian, H. and Zhang, L. (2011). Expression of CYP2E1 increases oxidative stress and induces apoptosis of cardiomyocytes in transgenic mice. *FEBS J.* 278, 1484-1492. 10.1111/j.1742-4658.2011.08063.x 21352494

[DMM049424C90] Zhang, C., Wang, Y., Ge, Z., Lin, J., Liu, J., Yuan, X. and Lin, Z. (2018). GDF11 attenuated ANG II-induced hypertrophic cardiomyopathy and expression of ANP, BNP and Beta-MHC through down- regulating CCL11 in mice. *Curr. Mol. Med.* 18, 661-671. 10.2174/1566524019666190204112753 30714521

[DMM049424C91] Zhao, J., Zhang, L., Lu, A., Han, Y., Colangelo, D., Bukata, C., Scibetta, A., Yousefzadeh, M. J., Li, X., Gurkar, A. U. et al. (2020). ATM is a key driver of NF-κB-dependent DNA-damage-induced senescence, stem cell dysfunction and aging. *Aging (Albany NY)* 12, 4688-4710. 10.18632/aging.102863 32201398 PMC7138542

[DMM049424C92] Zhou, Y., Duan, S., Zhou, Y., Yu, S., Wu, J., Wu, X., Zhao, J. and Zhao, Y. (2015). Sulfiredoxin-1 attenuates oxidative stress via Nrf2/ARE pathway and 2-Cys Prdxs after oxygen-glucose deprivation in astrocytes. *J. Mol. Neurosci.* 55, 941-950. 10.1007/s12031-014-0449-6 25407820

