# Supplementary material for: Inhibition of activin A receptor signalling attenuates age-related pathological cardiac remodelling
Source: Dis Model Mech. 2022 May 9;15(5):dmm049424. doi: 10.1242/dmm.049424 (PMC9118092; doi:10.1242/dmm.049424)
Supplement: Supplementary information [file dmm-15-049424-s1.pdf]

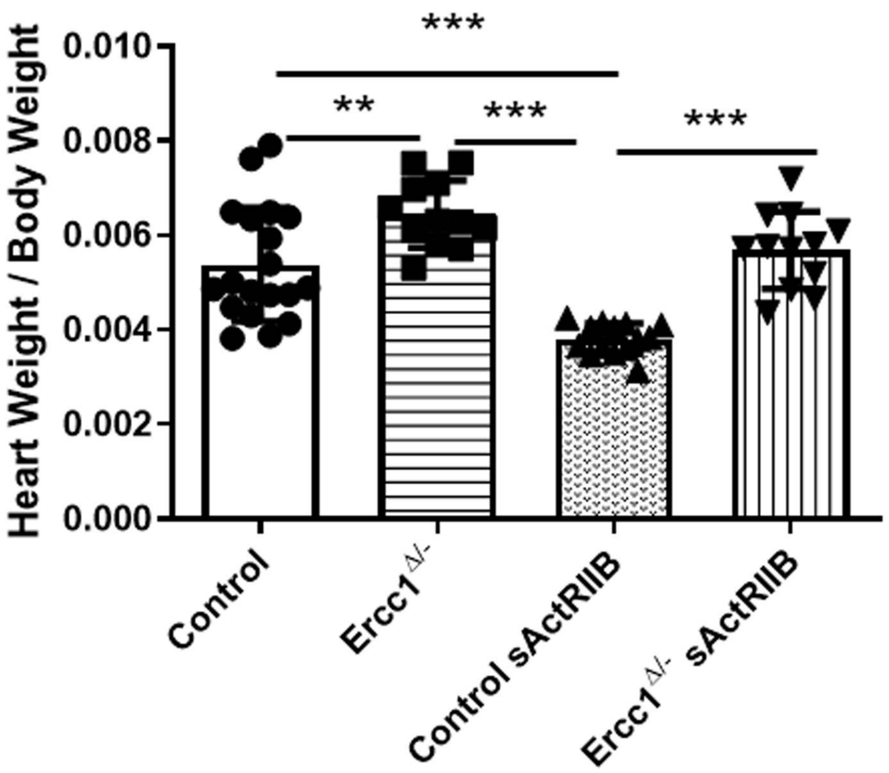

Fig. S1. Heart weight to body weight ratio increases in *Ercc1*$^{\Delta/-}$ progeroid mice. Heart weight / body weight ratio was measured from heart weight and tibia length measurement from control (n=19), *Ercc1*$^{\Delta/-}$ sActRIIB, *Ercc1*$^{\Delta/-}$ mice (n=12) and control sActRIIB mice (n=14) aged of 16 weeks. The heart weight of the mice was normalized to the body weight in order to compare the heart size (hypertrophy or atrophy) between the different experimental groups. One-way ANOVA parametric test was realised followed by a Tukey post-hoc test to compare the mean for different groups. Results are expressed as mean of heart weight / body weight ratio ± S.D. control *vs Ercc1*$^{\Delta/-}$, p=0.008757; control *vs* control sActRIIB, p=0.000028; *Ercc1*$^{\Delta/-}$ *vs Ercc1*$^{\Delta/-}$ sActRIIB, p=0.155720; *Ercc1*$^{\Delta/-}$ *vs* control sActRIIB, p<0.000001; control sActRIIB *vs Ercc1*$^{\Delta/-}$ sActRIIB, p=0.000008.

Disease Models & Mechanisms • Supplementary information

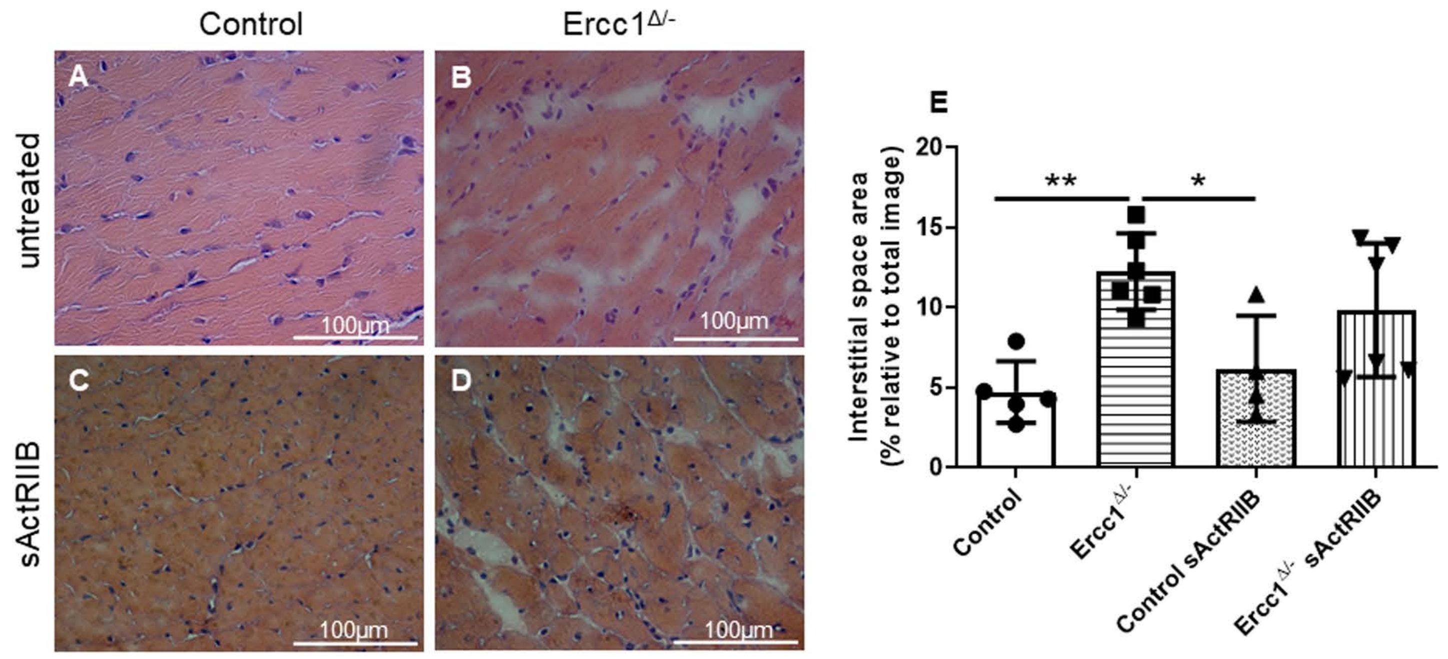

Fig. S2. Tissue interstitial space was increased in *Ercc1$^{\Delta/-}$* progeroid mouse hearts. (A-E) Haematoxylin and eosin staining highlights the cardiac interstitial spaces from control and *Ercc1* mice. **(A-D)** Frozen cardiac sections from mice control (n=5), *Ercc1$^{\Delta/-}$* untreated (n=6) (upper panels) and control, *Ercc1$^{\Delta/-}$* sActRIIB-treated mice (n=4, n=6) (lower panels) aged of 16 weeks. (E) Interstitial space area was quantified with ImageJ software, expressed as a percentage relative to the total picture are then averaged and pooled for each animal. One-way ANOVA parametric test was realised followed by a Tukey post-hoc test to compare the mean from different groups. Results are expressed as mean of interstitial area ± S.D. control *vs* *Ercc1$^{\Delta/-}$*, p=0.004584; control *vs* control sActRIIB, p=0.897504; *Ercc1$^{\Delta/-}$* *vs* control sActRIIB, p=0.034091; *Ercc1$^{\Delta/-}$* vs *Ercc1$^{\Delta/-}$* sActRIIB, p=0.546664.

Disease Models & Mechanisms • Supplementary information

Table S1. Overlapping genes between alternatively spliced genes and differentially expressed genes from the RNAseq analysis.

Target genes which are both differentially expressed and alternatively spliced		
Control – $Ercc1^{\Delta/-}$	Control – Control sActRIIB-treated	$Ercc1^{\Delta/-}$ sActRIIB-treated – Control sActRIIB-treated
ACKR4	ADAM23	LRP2BP
CIART	MT-ND4L	HACD4
TEF	UCKL1OS	ADAMTS2
TSC22D3	PLEKHH1	KIF20A
UCKL1OS	GM10222	NUSAP1
SNRPN	PRG4	CDKN1A
DMD	UCK2	LOCKD
GM11696	RCAN1	ACKR4
UCK2	SYNPO2L	FANCI
HSPH1	ALPL	KIF4
HSP90AA1	PTGDS	MYL1
RCAN1		CCNA2
ANO10		CDCA8
MYL1		ANO10
GM38393		ERCC1
SNHG14		GM45552
MID1IP1		MSL3
USP2		MT-ND4L
IRF7		MT-TI
FAM122B		FOXM1
2610028H24RIK		MXD3
DBP		GM10222
		GM12295
		C2
		CFB
		SHISA3
		GPSM2
		CD72

Column 1 shows 22 overlapping genes from the pairwise comparison between control and *Ercc1*$^{\Delta/-}$ progeroid mice. The middle column shows 11 overlapping genes from the pairwise comparison between control and sActRIIB treated mice. The right column shows 28 overlapping genes from the pairwise comparison between treated control and treated *Ercc1*$^{\Delta/-}$ mice.

Disease Models & Mechanisms • Supplementary information
